# Polyunsaturated fatty acids and p38-MAPK link metabolic reprogramming to cytoprotective gene expression during dietary restriction

**DOI:** 10.1038/s41467-020-18690-4

**Published:** 2020-09-25

**Authors:** Manish Chamoli, Anita Goyala, Syed Shamsh Tabrez, Atif Ahmed Siddiqui, Anupama Singh, Adam Antebi, Gordon J. Lithgow, Jennifer L. Watts, Arnab Mukhopadhyay

**Affiliations:** 1 Molecular Aging Laboratory, National Institute of Immunology, Aruna Asaf Ali Marg, New Delhi, 110067 India; 2 Buck Institute for Research on Aging, 8001 Redwood Blvd., Novato, CA 94945 USA; 3 Department of Molecular Genetics of Ageing, Max Planck Institute for Biology of Ageing, Cologne, 50931 Germany; 4 Cologne Excellence Cluster on Cellular Stress Responses in Aging Associated Diseases, University of Cologne, Cologne, 50931 Germany; 5 School of Molecular Biosciences, Washington State University, Pullman, WA 99164-7520 USA

**Keywords:** Cell signalling, Lipid signalling, Nutrient signalling, Gene expression, Gene regulation

## Abstract

The metabolic state of an organism instructs gene expression modalities, leading to changes in complex life history traits, such as longevity. Dietary restriction (DR), which positively affects health and life span across species, leads to metabolic reprogramming that enhances utilisation of fatty acids for energy generation. One direct consequence of this metabolic shift is the upregulation of cytoprotective (CyTP) genes categorized in the Gene Ontology (GO) term of “Xenobiotic Detoxification Program” (XDP). How an organism senses metabolic changes during nutritional stress to alter gene expression programs is less known. Here, using a genetic model of DR, we show that the levels of polyunsaturated fatty acids (PUFAs), especially linoleic acid (LA) and eicosapentaenoic acid (EPA), are increased following DR and these PUFAs are able to activate the CyTP genes. This activation of CyTP genes is mediated by the conserved p38 mitogen-activated protein kinase (p38-MAPK) pathway. Consequently, genes of the PUFA biosynthesis and p38-MAPK pathway are required for multiple paradigms of DR-mediated longevity, suggesting conservation of mechanism. Thus, our study shows that PUFAs and p38-MAPK pathway function downstream of DR to help communicate the metabolic state of an organism to regulate expression of CyTP genes, ensuring extended life span.

## Abstract (web summary)

Metabolic reprogramming during Dietary Restriction (DR) activates cytoprotective gene expression. Here the authors show that PUFAs generated during DR signal via the p38-MAPK pathway to enhance cytoprotective gene expression, contributing to increased longevity.

## Introduction

Dietary restriction (DR) is the only non-genetic manipulation that extends life span and delays age-associated diseases in most model systems. In non-human primates, DR has been shown to delay age-onset type 2 diabetes, cardiovascular diseases and loss of cognitive abilities^1^. Although the molecular mechanisms of DR are less clear, it is associated with dramatic reprogramming of metabolism that shifts from fat storage to enhanced fatty acid utilization, influencing cellular stress signaling pathways and reactive oxygen species (ROS) production^1–7^. Using *C. elegans*, we have shown that this metabolic reprogramming leads to the upregulation of the cytoprotective (CyTP) genes categorized to function in the xenobiotic detoxification program (XDP), based on Gene Ontology (GO) terms, and this coupling is essential for life-span extension during DR^3^. Knocking down either the central regulator of fatty acid metabolism (NHR-49) or transcription factors (NHR-8 or PHA-4) required for the expression of CyTP genes suppresses DR-induced longevity^3^. However, the signaling events that lead to activation of CyTP genes following increased fatty acid oxidation are not known.

Mitogen-activated protein kinases (MAPK) constitute the evolutionarily conserved signaling cascades that transduce signals, generated by extracellular stimuli, to the nucleus. The three well-characterized subfamilies of MAP kinases which have representatives in all eukaryotes include ERKs (extracellular signal-regulated kinases), SAPK/JNKs (stress-activated protein kinases/c-Jun N-terminal kinases) and p38-MAPK^8^. The p38-MAPK pathway has been implicated in diverse cellular responses including inflammation, stress response, cell cycle, apoptosis, development, differentiation, senescence, and tumorigenesis^9–12^. In *C. elegans*, the p38-MAPK pathway is an important regulator of innate immunity, oxidative stress resistance, neuronal development as well as longevity^13–17^. Upon exposure to pathogens or oxidative stress, a cascade of signaling events involving NSY-1 (MAPKKK)-SEK-1 (MAPKK) activates the downstream effector kinase PMK-1/p38-MAPK. This in turn regulates the transcriptional activation of innate immunity or ROS detoxification genes, respectively^14,16^. Despite its known role in regulating longevity downstream of the insulin-like signaling^15,18^, the p38-MAPK pathway’s role in DR-mediated longevity has not been clearly elucidated.

The present study defines the critical role of the p38-MAPK pathway in ensuring DR-mediated longevity. Using three different paradigms of DR in worms, we show that core components of the p38-MAPK pathway are essential for DR-induced life-span extension. In a genetic model of DR, we show that p38-MAPK pathway does not mediate the DR-induced metabolic shift towards fatty acid oxidation but is required for transcriptional upregulation of the CyTP genes. Further, we show that the levels of polyunsaturated fatty acids (PUFAs), especially linoleic acid (LA) and eicosapentaenoic acid (EPA) increase during DR and that the life span of DR worms is dependent on specific PUFA biosynthesis genes. Importantly, LA and EPA are able to independently activate the CyTP genes, in a p38-MAPK-dependent manner. Overall, our study suggests that the p38-MAPK pathway may respond to products of metabolic reprogramming associated with DR to upregulate CyTP genes. Considering the conserved nature of DR and the p38-MAPK pathway, such coupling may underlie the beneficial role of DR in different dietary paradigms in worms as well as in higher organisms.

## Results

### *C. elegans* p38-MAPK signaling mediates DR-induced life-span extension

**Fig. 1:**
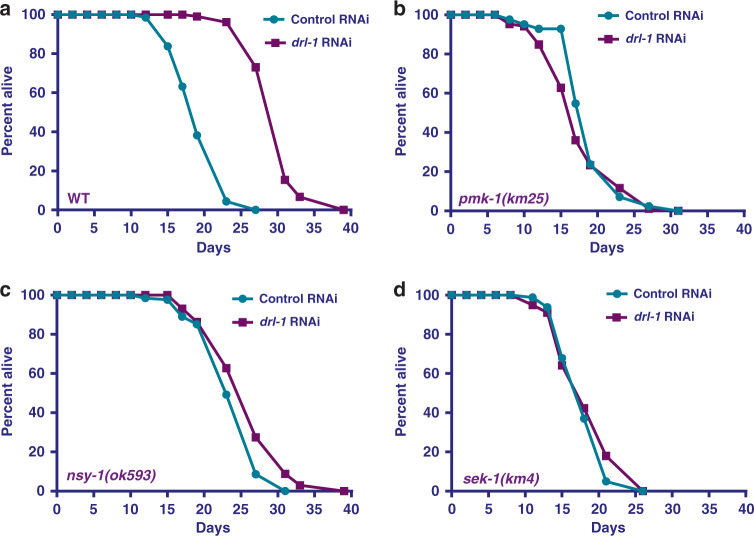
DR-like condition initiated by *drl-1* knockdown requires p38-MAPK pathway components. **a** The life span of wild-type (WT) worms are extended when *drl-1* is knocked down using RNAi. **b**–**d** The extended life span on *drl-1* knockdown is dependent on *nsy-1*, *sek-1*, and *pmk-1*. No life-span extension is observed when *drl-1* is knocked down in *pmk-1(km25)* (**b**), *nsy-1(ok593)* (**c**), or *sek-1(km4)* (**d**). Life span was performed at 20 °C and life-span summary data is provided in Supplementary Table 1.

In *C. elegans*, DR may be implemented using genetic mimics as well as by non-genetic interventions. We have shown that RNAi inhibition of a *C. elegans* ortholog of the human NEK2 (NIMA-related kinase 2) gene, *drl-1*, mimics a DR-like condition and extends life span, without altering food intake^3^. To identify additional components of the DRL-1 signaling, we screened for the MAPK mutants that failed to show life span extension upon exposure to *drl-1* RNAi. We found *drl-1* RNAi increased the life span of wild-type (WT) worms (Fig. 1a, also see Supplementary Table 1) but failed to extend the life span of *pmk-1(km25)* (Fig. 1b), a deletion mutant of the worm p38-MAPK ortholog. The PMK-1 P38-MAPK in *C. elegans* is activated by the upstream MAPKK, SEK-1 and the MAPKKK, NSY-1. We found that deletion mutants of *nsy-1* (Fig. 1c) and *sek-1* (Fig. 1d) kinases showed no life-span extension upon *drl-1* knockdown (KD), confirming the requirement of the p38-MAPK pathway in DRL-1 signaling. We ruled out the possibility of any defect in the RNAi machinery in these mutants by feeding embryonically lethal *pos-1* RNAi (Supplementary Fig. 1a); like wild-type, all p38-MAPK pathway mutant larvae failed to reach adulthood on *pos-1* RNAi. Also, we observed no changes in the pharyngeal pumping rate between *sek-1* and wild-type worms, confirming that inhibition of p38-MAPK signaling does not affect intake of RNAi-producing bacteria (Supplementary Fig. 1b). We further found that this effect on life-span extension of the *drl-1* model of DR is specific to p38-MAPK signaling, because the *drl-1* RNAi is able to increase life span of *jnk-1(gk7)* mutant, component of another prominent MAPK signaling pathway (Supplementary Fig. 1c). Also, inhibition of p38-MAPK signaling is not a limitation for life-span extension as reducing Insulin/IGF-1-like signaling by feeding *daf-2* RNAi leads to significantly increased life span of the *sek-1(km4)* mutant (Supplementary Fig. 1d).

**Fig. 2:**
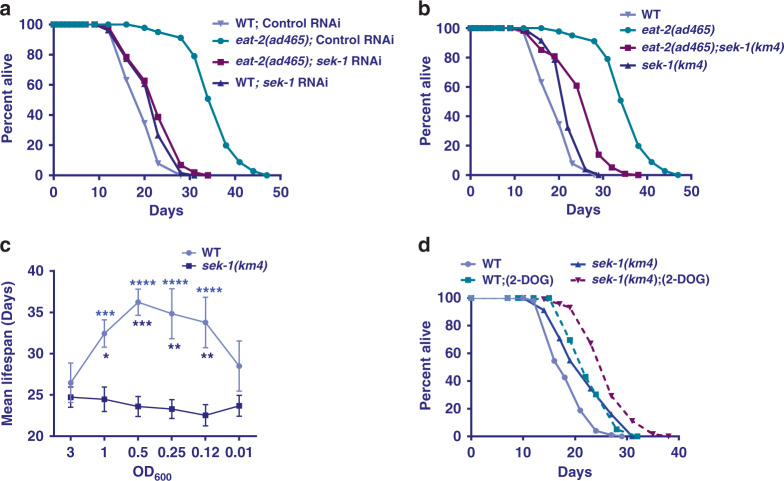
Other paradigms of DR also require p38-MAPK pathway components. **a** The life span of *eat-2(ad465)* is suppressed by *sek-1* RNAi. **b** The increased life span of *eat-2(ad465)* is partially abrogated in *eat-2(ad465);sek-1(km4)*. The above life spans were performed concurrently. **c** In bacterial dilution-induced DR (BDR), decreasing concentration of bacterial food produces a bell-shaped curve in WT. However, in *sek-1(km4)*, no life-span extension is observed at any of the dilutions. Bacterial culture of OD_600_ = 3.0 was the starting concentration for serial dilution. The increase in life span when WT was maintained at OD_600_ = 1.0, 0.5, 0.25, or 0.125 was significant as compared to OD_600_ = 3.0 (purple stars); the difference in case of *sek-1(km4)* was not. Two-way ANOVA-Tukey’s multiple comparisons test. ***P = 0.0005, ****P ≤ 0.0001. Also, the average life spans of WT at dilutions OD_600_ = 1.0, 0.5, 0.25, and 0.125 were significantly different from *sek-1(km4)* (dark blue stars). Two-way ANOVA-Sidak multiple comparisons test. *P = 0.0472, **P = 0.0015 (for OD_600_ 0.25), **P = 0.0021 (for OD_600_ 0.125), ***P = 0.0005. *n* = 4 independent experiments. Data are presented as mean values +/− SEM. **d** The life span of WT as well as *sek-1(km4)* is extended to the same extent when the worms were grown on 2-deoxyglucose (2-DOG). Life span was performed at 20 °C and life-span summary data is provided in Supplementary Table 1.

The *eat-2* mutants represent a well-established genetic model of DR in *C. elegans*^19^. We asked whether the p38-MAPK signaling is also required for the increased life span in this paradigm of DR. We found that *sek-1* RNAi significantly suppressed the increased life span of *eat-2(ad465)*, without affecting WT life span (Fig. 2a). Similarly, the life span of *eat-2(ad465);sek-1(km4)* double mutant is significantly lower than *eat-2(ad465)*, suggesting a genetic dependence (Fig. 2b). These results confirm that genetic DR regimes induced by *drl-1* RNAi and *eat-2* mutant require a downstream p38-MAPK signaling for life-span extension.

Apart from the genetic models described above, DR in *C. elegans* can also be implemented by serially diluting the bacterial feed (Bacterial Dilution Regime, BDR)^20,21^. Alternatively, feeding glucose analog, 2-deoxyglucose (2-DOG) has been shown to increase life span and mimic DR conditions in worms^22^. We therefore asked whether the p38-MAPK pathway is required for these non-genetic modes of DR. The BDR in WT worms generates a typical bell-shaped curve when average life span is plotted against the bacterial dilutions, showing a consistent increase in life span until an optimum DR threshold is reached. We observed that *sek-1(km4)* mutants undergoing BDR (Fig. 2c) failed to show any increase in life span when subjected to lower bacterial concentrations, confirming a complete dependence on the p38-MAPK pathway for longevity assurance. In contrast to this, 2-DOG induced DR does not require the p38-MAPK pathway as it was able to increase life span of the *sek-1(km4)* mutant (Fig. 2d). Together, we show that p38-MAPK is required for life-span extension by certain DR regimes but not others.

### p38-MAPK signaling is not required for DR-induced primary metabolic changes

**Fig. 3:**
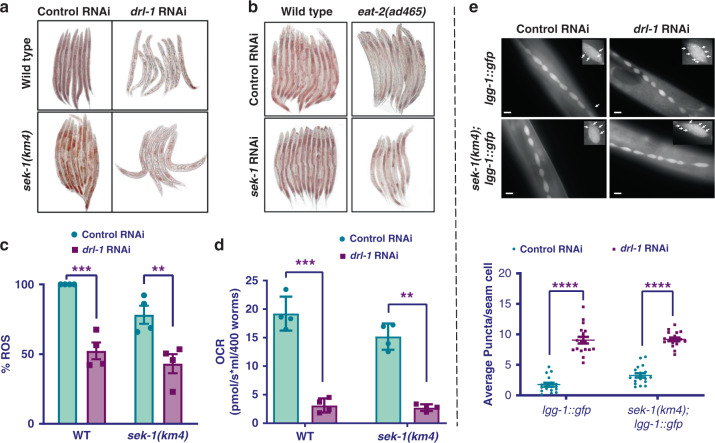
Metabolic reprogramming associated with DR does not require p38-MAPK. **a** Knocking down *drl-1* produces a DR-like phenotype in both WT as well as *sek-1(km4)*, resulting in depletion of stored fat to similar extent. Quantified data is provided in Supplementary Fig. 2a. One of three independent experiments is shown. **b** Depletion of stored fat during DR, as seen in *eat-2(ad465)*, is independent of *sek-1*. The *sek-1* RNAi did not significantly change the low fat of *eat-2(ad465)*. Quantified data is provided in Supplementary Fig. 2b. One of three independent experiments is shown. Multiple overlapping images were acquired at ×100 magnification to cover the entire worm body and stitched together to generate a contiguous image. **c** Knockdown of *drl-1* decreased ROS levels to similar extent in WT and *sek-1(km4)*, as determined by DCFDA assay. Data are presented as mean values ± SEM. *n* = 4 independent experiments. Two-way ANOVA-Sidak’s multiple comparisons test, ***P = 0.0004, **P = 0.0050. **d** Oxygen consumption rate (OCR) measured using Oroboros O2K shows similar respiratory rates in WT and *sek-1(km4)*. Data are presented as mean values ± SEM. *n* = 4 independent experiments. Unpaired two-tailed *t* test, **P = 0.0011, ***P = 0.0006. **e** Autophagy, as determined by puncta formation in the seam cells of a LGG-1::GFP-expressing strain (upper panel), was increased in both WT as well as in *sek-1(km4)* when *drl-1* was knocked down by RNAi. Representative images (upper panel) and quantification (lower panel) from one of two independent experiments shown. Experiments performed at 20 °C. *n* = 18 worms. Data are presented as mean values ± SEM. Two-way ANOVA-Sidak’s multiple comparisons test, ****P ≤ 0.0001. Scale bar = 10 μm. Experiments were performed at 20 °C. Source data are provided as a Source data file.

Metabolic changes during DR in *C. elegans* are generally associated with lowering of fat content, reduction in respiration rate, ROS production, and enhanced autophagy^3^. These changes are essential for increased longevity of the organism. Hence, we asked if any of these important cellular changes are mediated by the p38-MAPK signaling. We tested fat content of *drl-1* RNAi-fed wild-type and *sek-1(km4)* worms after staining them with Oil Red O stain and found that the fat content was significantly decreased for both strains (Fig. 3a, Supplementary Fig. 2a, related to Fig. 3a). Similarly, we also observed that there was no change in the fat content of *eat-2(ad465)* when p38-MAPK signaling was inhibited by either feeding *sek-1* RNAi (Fig. 3b, Supplementary Fig. 2b, related to Fig. 3b) or on crossing with *sek-1(km4)* (Supplementary Fig. 2c). These observations suggest that p38-MAPK signaling is not required for DR-induced changes in fat reserves. We also quantified the ROS levels in *drl-1* RNAi-treated wild-type and *sek-1(km4)* mutant worms by DCFDA assay and found similar decreases in both strains (Fig. 3c). In addition, *drl-1* knockdown worms exhibited lower respiratory rate (Fig. 3d) and higher autophagy that were not dependent on the p38-MAPK pathway (Fig. 3e). We also found that the *eat-2(ad465)* worms have similar trends in respiratory rate (Supplementary Fig. 3a) and autophagy (Supplementary Fig. 3b). All together, these results show that p38-MAPK signaling is not required for primary metabolic changes induced by genetic DR regimes.

### Transcriptional upregulation of CyTP genes during DR-like state requires p38-MAPK signaling

**Fig. 4:**
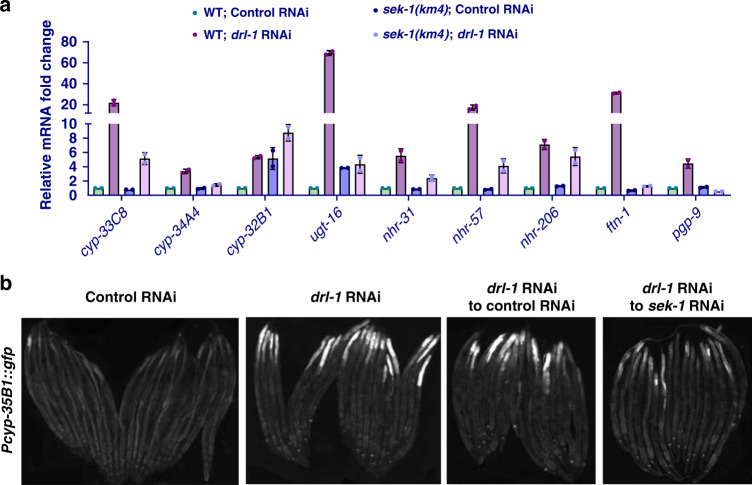
CyTP genes are upregulated during DR-like condition in a p38-MAPK-dependent manner. **a** Quantitative reverse transcriptase PCR (QRT-PCR) analysis shows that many Cytoprotective genes (CyTP) genes, of the GO category “xenobiotic detoxification pathway” (XDP), that are upregulated when *drl-1* is knocked down in WT, but failed to do so to the same extent in *sek-1(km4)*. Expression levels are normalized to *actin*. *n* = 2 biologically independent samples. Data are presented as mean values ± std. dev. **b** The expression of *Pcyp-35B1*::*gfp* is enhanced at YA stage when *drl-1* is knocked down using RNAi, compared to control RNAi. However, the upregulation is abrogated if *sek-1* is also knocked down by RNAi. Representative images from one of three independent experiments shown. Multiple overlapping images were acquired at ×100 magnification to cover the entire worm body and stitched together to generate a contiguous image. Experiments performed at 20 °C. Source data are provided as a Source data file.

We have earlier reported that metabolic reprogramming during DR leads to increased expression of CyTP XDP genes^3^, possibly to detoxify lipophilic endotoxins generated during increased fatty acid oxidation or to catalyze reactions to generate PUFA derivatives that are important signaling intermediates. Since the above results show that metabolic reprogramming during DR does not require p38-MAPK signaling, we next asked whether it mediates transcriptional upregulation of CyTP genes. For this, we compared the mRNA levels of a few selected, annotated Phase-I and Phase-II detoxification genes in wild-type and *sek-1(km4)* worms grown on control or *drl-1* RNAi. We found that expression of these genes increased on *drl-1* RNAi, as reported earlier^3^. Interestingly, in *sek-1(km4)* the expression of several of these genes is deregulated and *drl-1* knockdown could not increase their expression to the same extent as that in wild-type (Fig. 4a). We also validated the *sek-1* dependence using a transcriptional GFP reporter of the *cyp-35B1* gene (*Pcyp-35B1*::*gfp*) (Fig. 4b). The expression of GFP increased when worms were exposed to *drl-1* RNAi; however, expression was suppressed upon simultaneous introduction of *sek-1* RNAi. We confirmed that the *sek-1* RNAi led to dramatic decrease in the levels of activated p38-MAPK/PMK-1, without affecting its levels (Supplementary Fig. 4). These results suggest that p38-MAPK pathway may regulate DR-induced longevity by inducing CyTP genes, in response to products of the reprogrammed metabolism (e.g., fatty acids or their derivatives). When the pathway is abrogated, the expression of these genes is deregulated and they become recalcitrant to context-specific activation during DR.

### PUFAs generated during genetic DR regimes upregulate CyTP through p38-MAPK signaling

**Fig. 5:**
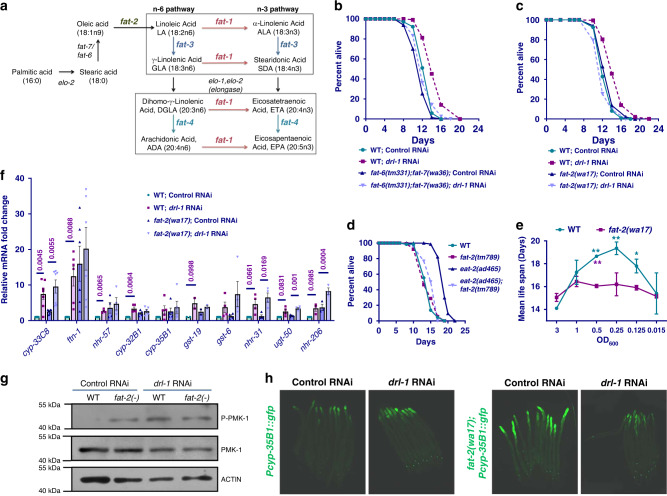
PUFA biosynthesis genes are required for *drl-1*-mediated life-span extension. **a** Schematic representation of the PUFA biosynthesis pathway in *C. elegans*. **b**, **c** Life span is extended when *drl-1* is knocked down in WT, but not to the same extent in *fat-6(tm331);fat-7(wa36)* (**b**) or *fat-2(wa17)* (**c**). **d** Life span of *eat-2(ad465)* is suppressed in *eat-2(ad465);fat-2(tm789)*. **e** In bacterial dilution-induced DR (BDR), *fat-2(wa17)* failed to produce a bell-shaped curve like WT. Bacterial culture of OD_600_ = 3.0 was the starting concentration for serial dilution. The increase in life span when WT was maintained at OD_600_ = 0.5, 0.25, or 0.125 was significant as compared to OD_600_ = 3.0 (green stars); the difference in case of *fat-2(wa17)* was not. Two-way ANOVA-Tukey’s multiple comparisons test. *P = 0.0346, **P = 0.0087 (for OD_600_ 0.5), **P = 0.0029 (for OD_600_ 0.25). The average life spans of WT at dilutions OD_600_ = 0.5 was significantly different from *fat-2(wa17)* (purple stars). Unpaired two-tailed *t* test. **P = 0.0036. Life spans were performed at 25 °C. Data presented as mean life span ± SEM. *n* = 2 biologically independent replicates. Life-span summary is provided in Supplementary Table 1. **f** The expression of selected CyTP genes are upregulated when *drl-1* is knocked down. The expression of several of them are dependent on *fat-2*, as determined by QRT-PCR. Expression levels were normalized to *actin*. *n* = 7 (*cyp-33C8)*, *n* = 6 (*ftn-1)*, *n* = 5 (*cyp-32B1, cyp-35B1*), *n* = 4 (*gst-6, nhr-31, ugt-50*), *n* = 3 (*nhr-206*), or *n* = 2 (*gst-19*) biologically independent samples. Data are presented as mean values ± SEM. Unpaired two-tailed *t* test. **g** Western blot analysis of WT and *fat-2(wa17)* showing that *drl-1* RNAi upregulates phospho-PMK-1. The levels of phospho-PMK-1 is already upregulated in *fat-2(-)* and is not further upregulated when *drl-1* is knocked down. One of two independent experiments shown. **h** The expression of *Pcyp-35B1*::*gfp* is enhanced at YA stage when *drl-1* is knocked down using RNAi, compared to control RNAi. However, *fat-2(wa17);Pcyp-35B1::gfp* already has elevated expression of GFP, and *drl-1* RNAi failed to further increase it. Representative images from one of three independent experiments shown. Multiple overlapping images were acquired at ×100 magnification to cover the entire worm body and stitched together to generate a contiguous image. Source data are provided as a Source data file. All experiments were performed at 25 °C.

Since the *drl-1* KD-induced CyTP gene upregulation is dependent on NHR-49^3^ as well as p38-MAPK signaling (this study), we explored the role of PUFAs in this process. The reason behind this is NHR-49, which apart from regulating the fatty acid oxidation genes, also modulates expression of genes encoding the Δ-9 desaturase enzymes FAT-5, FAT-6, and FAT-7^23^. These enzymes are essential for biosynthesis of PUFAs (Fig. 5a). In addition, previously published reports have found that the levels of PUFA increase during nutrient stress^24^ and various PUFAs have been shown to be important for maintaining the activity of the p38-MAPK^25^. We, therefore, hypothesized that the enhanced transcription of CyTP genes via the p38-MAPK pathway during DR may be due to the increased levels of PUFAs. We investigated this hypothesis by genetic and biochemical approaches using mutants defective in PUFA synthesis and GC-MS analysis to directly measure levels of PUFAs, respectively. We first performed *drl-1* RNAi life-span analysis in mutants defective in PUFA biosynthesis. We used *fat-6;fat-7* double mutant and *fat-2* mutant lacking Δ9- and Δ12-desaturase activities, respectively; consequently, these mutants are deficient in downstream PUFA biosynthesis^26–28^ but may accumulate monounsaturated oleic acid (OA) or saturated stearic acid (SA) and palmitic acid (PA) (Fig. 5a). We found that *drl-1* RNAi failed to increase life span in these mutants (Fig. 5b, c) highlighting an essential role of PUFAs in *drl-1* KD-induced DR longevity. Similarly, in the *eat-2(ad465)*, knocking out *fat-2* led to suppression of life span (Fig. 5d). We also performed BDR assay with *fat-2(wa17)* and found that in the mutant, the mean life spans, when plotted against bacterial dilutions, failed to produce a bell-shaped curve, typically seen in WT (Fig. 5e). These results show that when PUFA biosynthesis is perturbed, genetic as well as non-genetic DR regimes fail to increase life span, suggesting an involvement of the downstream PUFAs or their derivatives in activating p38-MAPK during DR.

We next asked if the failure to increase life span following *drl-1* KD in the PUFA-defective mutants is possibly due to abrogated transcriptional regulation of CyTP genes. For that, we grew the *fat-2* mutant on control or *drl-1* RNAi and determined the mRNA expression of the CyTP genes (Fig. 5f). Interestingly, we found that the mRNA expression of several of the CyTP genes was already upregulated (significantly for *ftn-1*, *cyp-35B1*, *cyp-32B1*, *gst-19*, and *nhr-206*) in the *fat-2* mutant. This deregulation of CyTP genes is accompanied by the enhanced p38-MAPK activity, as determined by a phospho-PMK-1 (pPMK-1) western blot analysis (Fig. 5g). However, unlike in wild-type, *drl-1* RNAi failed to produce similar levels of upregulation in many of these CyTP genes in the *fat-2* mutants (Fig. 5f). Consistent with the observation associated with CyTP genes expression, *drl-1* RNAi also failed to further increase the p38-MAPK activity in *fat-2* mutants (Fig. 5g). The *fat-2(wa17);Pcyp-35B1*::*gfp* worms also showed increased expression compared to wild-type; *drl-1* RNAi failed to increase the *gfp* expression beyond this level (Fig. 5h).

Since p38-MAPK is ectopically activated in the *fat-2* mutant that blocks PUFA biosynthesis, it suggests that OA, SA or PA may also independently activate the CyTP genes, in a p38-MAPK-dependent manner. Indeed, we observed that OA, SA, and PA activated p38-MAPK as well as enhanced the expression of XDP reporter gene *Pcyp-35B1*::*gfp*, dependent on a functional p38-MAPK pathway (Supplementary Fig. 5a, b). These results suggest that p38-MAPK as well as the CyTP genes may be ectopically activated in *fat-2* mutant worms due to the accumulation of upstream fatty acids or their derivatives. As a result, they become recalcitrant to context-specific activation during DR and life-span benefits are suppressed. Altogether, these results highlight the important role of a balance between SFA, MUFA, and PUFA in regulation of CyTP genes downstream of the p38-MAPK pathway to regulate longevity during DR.

**Fig. 6:**
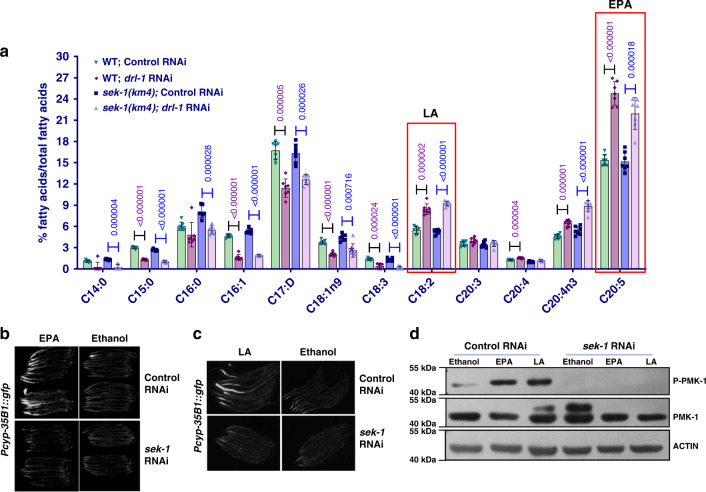
Eicosapentaenoic acid (EPA) and linoleic acid (LA) supplementation activates p38-MAPK. **a** GC-MS analysis revealed that PUFAs are differentially regulated when *drl-1* is knocked down in WT. The levels of these PUFAs change in a similar manner when *drl-1* is knocked down in *sek-1(km4)*. *n* = 7 biologically independent samples. Data are presented as mean values +/− std. dev. Unpaired two-tailed *t* test. **b**, **c** The *Pcyp-35B1*::*gfp* worms were grown on control or *sek-1* RNAi plates supplemented with EPA (**b**) or LA (**c**) since hatching. PUFA supplementation increased the expression of *Pcyp-35B1*::*gfp* when worms are grown on control RNAi but not on *sek-1* RNAi. Multiple overlapping images were acquired at 100X magnification to cover the entire worm body and stitched together to generate a contiguous image. One of two (LA) or three (EPA) independent experiments shown. **d** Western blot analysis of *Pcyp-35B1*::*gfp* grown on control or *sek-1* RNAi showing that EPA and LA supplementation upregulates phospho-PMK-1 levels, in a *sek-1-*dependent manner. One of three independent experiment shown. Source data are provided as a Source data file. All experiments were performed at 20 °C.

Further, to identify specific PUFA molecules that may be signaling to the p38-MAPK pathway under a DR-like condition, we quantified PUFAs in worms subjected to *drl-1* RNAi using GC-MS. We found that levels of several SFAs and MUFAs like C14:0, C15:0iso, C16:0, C16:1, and C17:Δ were significantly lower in *drl-1* RNAi worms compared to control RNAi (Fig. 6a). These fatty acids are abundant components of *C. elegans* triglyceride (TAGs)^29^. Lower levels of these fatty acids are consistent with our finding that *drl-1* RNAi results in lower fat stores in worms. In contrast, we found that the levels of PUFAs, including C18:2, C20:4, C20:4(n3), and C20:5 are significantly higher in *drl-1* RNAi-treated wild-type worms, supporting the important role of PUFAs during DR (Fig. 6a). As expected, we did not observe any major differences between the fatty acid profile of *sek-1* and wild-type worms treated with *drl-1* RNAi (Fig. 6a), strengthening our conclusion that p38-MAPK signaling may act downstream of fatty acid metabolism. We also performed GC-MS analysis of the *eat-2(ad465)* and compared it to that of wild-type (Supplementary Fig. 6) and found similar trend in most cases except for 18:2 fatty acid which may point to DR paradigm-specific differences.

Among the PUFAs detected in *drl-1* RNAi worms, levels of linoleic acid (C18:2n6; LA) and eicosapentaenoic acid (20:5n3; EPA) were significantly upregulated in wild-type grown on *drl-1* RNAi. So, we asked if these specific PUFAs could independently activate CyTP genes in a p38-MAPK signaling-dependent manner, bypassing the requirement of DR for metabolic reprogramming. We supplemented the reporter *Pcyp-35B1*::*gfp* worms with EPA or LA and found that the GFP expression was enhanced (Fig. 6b, c). Most importantly, this upregulation was dependent on p38-MAPK signaling as the increase was abrogated when *sek-1* was KD using RNAi (Fig. 6b, c). Further, supplementing EPA or LA were sufficient to activate PMK-1 as determined by phospho-western analysis (Fig. 6d), independent of *drl-1* signaling. Together, these experiments show a critical role of specific PUFAs in inducing CyTP genes via p38-MAPK pathway during DR.

**Fig. 7:**
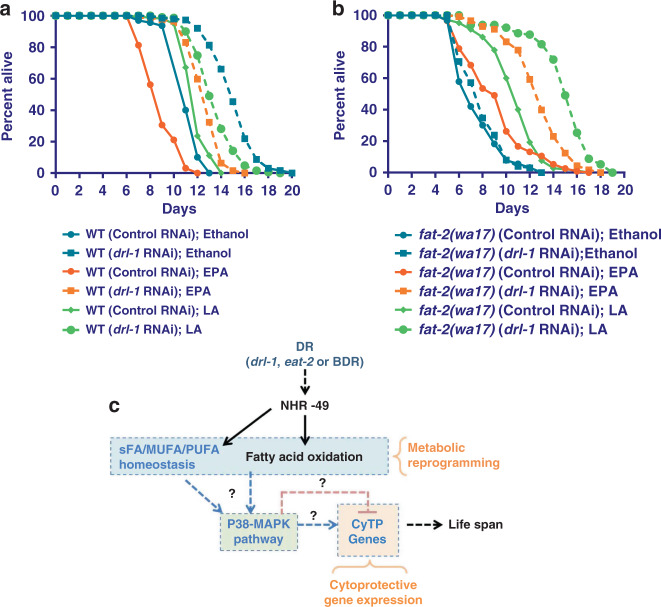
External supplementation of EPA or LA rescues life-span defects of *fat-2(wa17)*. **a**, **b** WT (**a**) or *fat-2(wa17)* (**b**) were grown on control or *drl-1* RNAi in the presence or absence of LA or EPA. In *fat-2(wa17)*, the *drl-1* RNAi failed to increase life span. However, the life span was rescued on addition of LA or EPA. Life span was performed at 25 °C and life-span summary data is provided in Supplementary Table 1. **c** A model showing that p38-MAPK functions downstream of different paradigms of DR to regulate gene expression and longevity, as discovered in this study. The ‘?’ denotes unanswered questions that evolve from the study. For example, How does sFA/MUFA/PUFAs or their derivatives activate p38-MAPK? What transcription factors function downstream of p38-MAPK to regulate CyTP genes? Source data are provided as a Source data file.

Next, we asked whether these specific PUFAs, LA and EPA, are sufficient to mediate the signaling events that results in increased life span of worms undergoing genetic DR. We grew wild-type or PUFA-defective *fat-2(wa17)* mutant on control or *drl-1* RNAi, in the presence or absence of LA or EPA and scored life span. The life span of WT was slightly increased by LA supplementation; but EPA decreased wild-type life span (Fig. 7a). However, in *fat-2(wa17)* mutant background, both LA and EPA increased life span. Importantly, while in *fat-2(wa17)* mutant, where *drl-1* KD failed to increase life span, life span was increased when the mutant was supplemented with LA or EPA (Fig. 7b).

Together, these experiments show that PUFA or its derivatives produced during DR activates p38-MAPK pathway and CyTP gene expression to mediate longevity benefits.

## Discussion

A metabolic shift to fatty acid utilization has been shown to play a prominent role in DR-mediated life-span extension in multiple model systems^2,6,30^. However, apart from supporting efficient energy utilization, we still lack a complete mechanistic understanding of the multitude of potential benefits that this shift endows. We have earlier shown that metabolic reprogramming to fatty acid oxidation during DR in *C. elegans* leads to a coupled activation of the cytoprotective genes, members of which are categorized as XDP genes according to Gene Ontology terms. This coupling is a requirement of DR-mediated life span extension in the two genetic DR models tested^3^. However, how the organism transmits the information of metabolic reprogramming to activate gene expression during DR was not known. In the present study, we show that PUFAs, especially LA and EPA, or possibly derivatives of it, which accumulate during DR, can act as potential signaling molecules to upregulate CyTP genes via the conserved p38-MAPK pathway (Fig. 7c). Previously, DR has been independently shown to upregulate EPA^31^ as well as activate XDP genes^32,33^ in mammals. Our study, using *C. elegans*, describes a previously unknown role of the PUFA-p38-MAPK signaling axis in cyto-protection and DR-mediated longevity assurance. Although, we have mostly used a genetic model of DR in our study, conservation of the components suggest that similar mechanisms may drive pro-longevity effects in other DR paradigms in *C. elegans* as well as in other species.

In *C. elegans*, metabolic reprogramming during nutrient deprivation is under the transcriptional control of NHR-49 that regulates the expression of both beta-oxidation as well as PUFA biosynthetic genes^23,34^. The lipid molecules may act as a double-edged sword. While metabolic shift to fatty acid oxidation in worms leads to efficient energy utilization during DR as well as reduced ROS production^3,35,36^, an imbalance may be detrimental to the cells. Lipid molecules in the cellular milieu are prone to oxidative modifications that are hazardous. PUFA molecules are extremely prone to peroxidation, whereby free radicals attack carbon–carbon double bonds in the lipids^37,38^. PUFA peroxides such as malondialdehydes and 4-hydroxynonenal (4-HNE), are the most extensively-studied lipid peroxidation end-product and are damaging to cells. Interestingly, 4-HNE shows physiological and protective function as a signaling molecule that stimulates gene expression. However, it also shows cytotoxicity by inhibiting gene expression and promoting the development and progression of different pathological states^39^. Oxidized omega-3 PUFA has been shown to induce oxidative stress and inflammation^40^. In contrast, deuterated PUFAs retard lipid peroxidation and is capable of regulating oxidative stress, aging, and providing life-span benefits in *C. elegans*^41^. In addition, PUFAs are associated with lipid membranes from where they are released by phospholipase A2 and undergo processing to many biologically active signaling molecules in mammals like prostaglandins (PGs), prostacyclins and thromboxanes (TXs), lipoxins (LXs) and leukotrienes (LTs), protectins, marensins, and resolvins, epoxyeicosatrienoic acids (EETs), and hydroxyeicosatetraenoic acids (HETEs) that have pro- and anti-apoptotic properties, play important roles in inflammation, modulate the immune responses, and other unknown processes^42^. Although *C. elegans* lack cyclooxygenase orthologs, several F-series prostaglandins are synthesized, using a cyclooxygenase-independent pathway, from C20 PUFA precursors in the female germline that help to attract sperm to oocytes^29^. Thus, an elevated level of various PUFAs (like LA or EPA) as observed on *drl-1* RNAi and in the *eat-2* mutant may result in increased peroxidation that are potentially detrimental to the organism, or can lead to dysregulation of key signaling events, if not properly contained by detoxification. These may be perceived as danger or stress signals that could activate the cytoprotective processes through p38-MAPK pathway. The xenobiotic detoxification system is capable of detoxifying a plethora of lipotoxins including steroids, eicosanoids, and other fatty acid derivatives that may constitute the lipophilic endotoxins that are byproducts of increased fatty acid metabolism. DR has indeed been shown to activate the phase-II detoxification system both at transcriptomic as well as biochemical level in mammals^32,33^. It may, however, be noted that while many of these cytoprotective genes are classified as XDP genes according to Gene Ontology analysis, they have other significant functions. For example, CYP-22A1 or DAF-9 produces a bile acid-like hormone that binds to DAF-12 receptor to control development and longevity^43–45^. Others have important function in fat storage regulation^46^ or directional sperm motility^47^. At the biochemical level, CYP-29A3 and CYP-33E2 are the major CYP isoforms that have been shown to be involved in EPA metabolism^48^ and may have important signaling roles. Together, they may contribute to cyto-protection and signaling required for enhanced longevity. So, there is merit in coupling metabolic reprogramming to activation of these cytoprotective machineries during DR through the stress-responsive p38-MAPK pathway.

We have shown here that DR or external supplementation of LA and EPA induce the p38-MAPK pathway, both in terms of increased pPMK-1 levels as well as increased CyTP gene expression. Previous studies have also shown that p38-MAPK pathway may be activated by PUFAs and other fatty acids under different physiological conditions. External supplementation experiments have shown that PUFAs have beneficial effects on life span and innate immunity by activating the p38-MAPK pathway in *C. elegans*^49^. The *fat-3* mutants defective in γ-linolenic acid (GLA) and stearidonic acid (SDA) biosynthesis have reduced basal activity of p38-MAPK that can be rescued by external supplementation of these lipids^25^. Arachidonic acid activates members of the mitogen-activated protein kinase superfamily in rabbit proximal tubule cells^4^, hepatocytes^50^, and human brain endothelial cells (HBECs)^51^. The MUFA oleic acid has been shown to activate p38-MAPK in hepatocytes^52^ and in rat hepatoma dRLh-84 cells^53^. In vascular endothelial cells, LA activates p38-MAPK within 10 min of treatment^54^. In primary hepatocytes, fasting-induced free FAs activate p38-MAPK providing a likely reason of this pathway acting as a metabolic sensor for increased PUFA and associated threat during DR^55^. Our current and previous studies, in combination, provides a similar context for activation of p38-MAPK and CyTP genes to counter stress, potentially generated by lipophilic endotoxins produced due to metabolic reprogramming.

However, the question remains about the mechanisms of activation of the p38-MAPK pathway by fatty acids during DR. Levels of omega-3 PUFAs have been shown to enhance sensitivity of TRPV channels by altering membrane fluidity and modulating Ca^2+^ levels^56^. Also, in *fat-3* mutants, diminished calcium response and associated avoidance response is rescued by 20-carbon omega-6 and omega-3 PUFAs^57^. In addition, TRP-like channels mediate upregulation of α-dicarbonyl detoxification system in a p38-MAPK-dependent manner^58^. Thus, it is tempting to speculate that increase in PUFA levels during DR maybe activating p38-MAPK pathway and associated CyTP genes by increasing sensitivity of TRP channels. PUFAs can also activate p38-MAPK through the generation of ROS, and supplementation of Vitamin E is able to abrogate this response^59^. However, activation through ROS seems counter intuitive, because DR reduces the level of these molecules. Further investigation is required to decipher the mechanisms of p38-MAPK activation by PUFAs during DR.

A delicate balance of SFA/MUFA/PUFA levels are maintained within the cell for optimal function of the structural and signaling elements. The toxic effects of PUFA imbalance is particularly apparent in the germline. Dietary supplementation of omega-6 PUFA-dihomogamma linolenic acid (DGLA, 30:3n6) results in sterility due to destruction of germ cells^60,61^. Strains that have higher detoxification capabilities, like the Insulin/IGF-1-like signaling receptor *daf-2* mutant worms are able to resist DGLA-induced germline sterility^61^. Since PUFAs are more likely to undergo peroxidation, which in turn increases ROS production, long-lived worms like *daf-2* mutants have increased MUFA with decreased PUFA levels^62^. External supplementation of MUFAs and PUFAs have shown opposite effects with the former suppressing and later sensitizing the cells to lipid peroxidation^38^. Dietary supplementation with fish oil increased levels of lipid peroxidation and induced shorter life span in *C. elegans*^63^. On the other hand, lower concentrations of omega-6 PUFAs activate autophagy and increase life span^24^. Since PUFAs may be rapidly turned over, it is possible that oxidized PUFAs are promptly eliminated from the system and worms that have active detoxification/elimination machinery may actually benefit even from increased PUFA levels, as seen in this study. The careful balancing of PUFA metabolism is also apparent in mammals where neural stem cell niche triglycerides are enriched in oleic acids and infusing oleic acid directly into brain ventricles is sufficient to inhibit neural stem cell proliferation^64^. Our GC-MS data shows that both *drl-1* KD and *eat-2* mutant worms are low in saturated fatty acids and MUFAs, while some PUFA levels are elevated, providing life-span benefits. However, a disbalance caused by *fat-2* mutation leads to deregulation of the p38-MAPK pathway, its associated gene expression as well as suppression of DR-mediated life span.

The *C. elegans* p38-MAPK pathway is involved in diverse aspects of cellular function, including development^65,66^, stress response^14,18^, innate immunity^15,25,67,68^, metabolism, and longevity^69,70^. In all these cases, when a component of the p38-MAPK pathway was mutated, the phenotype was adversely affected. For example, the worms deficient in p38-MAPK signaling were found to be susceptible to *Pseudomonas aeruginosa* infection^15,68^ and decline in p38-MAPK signaling was shown to cause immune senescence^67^. The p38-MAPK pathway mutants were susceptible to oxidative stress^14^. The increased life span of *daf-2* mutants^15^ and the food-type dependent life span of *flr-4* mutant are dependent on p38-MAPK pathway components^70^. In most of the cases, the activation of the p38-MAPK pathway correlates with the phenotype. For instance, the phosphorylation of PMK-1 is increased on oxidative stress^14^ and *flr-4* mutant worms have higher phospho-PMK-1 on *E. coli* HT115 bacteria^70^. Although the phosphorylation of PMK-1 has not been directly elucidated on challenge with pathogenic bacteria, susceptible worms have lower amount of pPMK-1^67,68^. In this study, we show that the p38-MAPK pathway is required for both genetic and non-genetic modes of DR, and in case of genetic DR, the pathway is activated and pPMK-1 levels are higher. In contrast to these observations, a recently published study by Wu et al. showed that although non-genetic modes of DR require p38-MAPK components, the phospho-PMK-1 levels are reduced under this condition^69^. These differences in observations may be attributed to the mode of DR implementation as well as the time-frame when the pPMK-1 westerns were performed. Interestingly, the DR paradigms used in our study that are dependent on the p38-MAPK, also show dependency on the FOXA transcription factor PHA-4 and not the FOXO transcription factor DAF-16^3,19,21^. On the other hand, the DR paradigm used in Wu et al. is dependent on FOXO/DAF-16. While these studies underscore the important contribution of the p38-MAPK pathway in DR-induced longevity, it will be important to extensively elucidate the underlying mechanisms in much greater details.

In this context, it may also be noted that unlike *drl-1* and *eat-2* genetic models, we did not find a requirement of p38-MAPK pathway in 2-DOG-mediated life-span extension. Different DR models are known to have distinct as well as complex overlapping mechanisms^71^. For example, in *drl-1* and *eat-2* models, ROS is inherently low, while 2-DOG-mediated life-span extension is mediated via the hormetic effects of ROS^3,22^. Despite these differences, *drl-1* KD worms, *eat-2* mutants as well as 2-DOG fed-DR models seems to have a similar downstream NHR-49-mediated metabolic shift that is required for life-span extension^3,72^. How or when DR is initiated may play an important role in activating the p38-MAPK pathway and downstream cytoprotective genes.

## Methods

### Strain maintenance

Unless mentioned otherwise, all strains were obtained from CGC Minnesota and maintained at 20 °C on standard Nematode Growth Media (NGM) seeded with *Escherichia coli* (OP50). The *E. coli* bacteria were grown overnight in Luria Bertani (LB) media at 37 °C, and 200 or 1000 µl of the culture was seeded on 60 or 90 mm NGM agar plates, respectively. The plates were set at room temperature for 2–3 days to allow the bacteria to grow. For all RNAi assays, the RNAi was initiated from eggs or synchronized L1s, unless mentioned explicitly. The strains used in the study are: N2 Bristol as wild-type, VC390 *nsy-1(ok593) II*, KU4 *sek-1(km4) X*, KU25 *pmk-1(km25) IV*, VC8 *jnk-1(gk7) IV*, DA465 *eat-2(ad465) II*, DA1116 *eat-2(ad1116) II*, CY573 *bvIs5[cyp-35B1p::GFP*+*gcy-7p::GFP]*, BX26 *fat-2(wa17) IV*, BX156 *fat-6(tm331) IV;fat-7(wa36) V*, DA2123 *adIs2122 [lgg-1p::GFP::lgg-1*+*rol-6(su1006)]*, *fat-2(tm789) IV*. The strains *eat-2(ad465);fat-2(tm789), eat-2(ad465) II;sek-1(km4) X*, and *sek-1(km4);adIs2122* were generated in the lab.

### RNAi lifespan

For performing life-span analysis, NGM RNAi plates (60-mm diameter) were prepared by supplementing NGM with 100 µg/ml ampicillin and 2 mM isopropyl β-d-1-thiogalactopyranoside (IPTG). RNAi bacteria were grown overnight at 37 °C in LB media containing 100 µg/ml ampicillin and 12.5 µg/ml tetracycline. The cultures were diluted next day (1:100 v/v) in LB containing 100 µg/ml ampicillin and grown at 37 °C till an OD_600_ of 0.6 was attained. The bacterial pellets were then resuspended in (1:10 v/v) 1X M9 buffer containing 1 mM IPTG and 100 µg/ml ampicillin. About 200 µl bacterial suspension was plated on the NGM RNAi plates and allowed to dry for 1 day.

Synchronized population of worms was obtained by sodium hypochlorite treatment of gravid adult hermaphrodite and eggs were allowed to hatch and grow on plates containing the respective RNAi bacteria. For synchronizing L1s, the eggs were hatched in 1X M9 for 16 h. Day-1 adult worms were then transferred to respective RNAi plates overlaid with 5-fluorodeoxyuridine (FUDR; final concentration of 100 µg/ml)^73^. Worms were scored as dead or alive by gently tapping them with a platinum wire every 2–3 days. Sick worms showing vulval bursting or worms that crawled to the sides/lid of the plates were censored. The *fat-2* mutants have been reported to show delayed development at 20 °C^27^, thus all the life spans involving *fat-2* mutants were performed at 25 °C.

### 2-DOG lifespan

For performing 2-DOG lifespan, NGM agar was supplemented with 2-DOG (Sigma-Aldrich, D8375) to a final concentration of 5 mM. Synchronized egg population (100–150) obtained by sodium hypochlorite treatment of gravid worms grown on *E. coli* OP50 was transferred to control RNAi plates without 2-DOG. At young adult (YA) stage, approximately 50 worms were transferred to the 2-DOG-containing or control plates, both overlaid with FUDR, in three technical replicates. Worms were then scored as live or dead every alternate day. Sick worms with ruptured vulva rupture were censored from the population.

### Bacterial dilution-induced DR (BDR) assay

The BDR assay was performed by preparing *E. coli* OP50-L4440 (for Fig. 2c) or HT115-L4440 (Fig. 5e) BDR media as described below. Using a platinum loop, *E. coli* was streaked on a LB agar plate and incubated at 37 °C for 12 h. From the plate, single bacterial colony was used to inoculate 200 ml LB in a 2-litre flask. Bacteria were allowed to grow at 37 °C for 12 h in an incubator shaker (Innova 42 incubator shaker, New Brunswick Scientific, Edison, NJ, USA) at 250 rpm. The bacterial cells were collected by centrifugation at 3215 × *g*, 4 °C for 10 min and resuspended in S-basal–cholesterol–antibiotics solution (cholesterol 5 µg/ml, carbenicillin 50 µg/ml, tetracycline 1 µg/ml, kanamycin 10 µg/ml). The desired bacterial dilution was measured by further diluting cells in S-basal–cholesterol–antibiotics solution and measuring OD_600_. These diluted bacterial solutions were stored at 4 °C for a maximum of 2 weeks. Synchronized egg population obtained by sodium hypochlorite treatment of well-fed gravid adult worms were allowed to grow on a 90 mm NGM plate seeded with OP50 bacteria till young adult age. At this point, FUDR (100 µg/ml) was added on plates to prevent any progeny development. Post 24 h, worms were transferred to 12-well cell-culture plate (30 worms/well) containing 1 ml of S-basal–cholesterol–antibiotics solution with FUDR (100 µg/ml). The plate was kept on a shaker maintained at 20 °C for 1 h to remove adhering bacteria. During this time, diluted bacterial suspension for the lifespan were added to the 12-well cell-culture plates (1 ml solution/well along with FUDR at 100 µg/ml). After 1 h, 10–12 worms/well were moved from the S-basal to the diluted bacterial suspension using a glass pipette connected to a P200 pipette. Worms were moved to fresh bacterial solutions with respective ODs every 3–4 days and scored for movement by prodding using a platinum wire. FUDR supplementation in diluted bacterial suspension was stopped after 8 days. Worms that did not respond to gentle prodding with a worm pick were scored as dead and removed. During experiments, plates were maintained at 20 °C for *sek-1(km4)* and 25 °C for *fat-2(wa17)* experiments, in an incubator shaker (Innova 42 incubator shaker, New Brunswick Scientific, Edison, NJ, USA) at rotation speed of 100 rpm. Range of OD_600_ used: 3.0, 1.0, 0.5, 0.25, 0.125, and 0.0156.

### Lethality assay

The *pos-1* gene codes for a CCCH-type zinc finger protein that is required for specification of germ cells, intestine, pharynx, and hypodermis^74^; knocking it down leads to larval lethality. Wild-type, *nsy-1, sek-1*, and *pmk-1* L4 animals were picked and maintained on control or *pos-1* RNAi-seeded plate till day-1 of adulthood and sacrificed. Eggs laid on the RNAi plates were scored for hatching. Percentage of eggs that failed to hatch or arrested early in development (embryonic lethal) were plotted on X-axis with Y-axis indicating the genotype.

### Quantification of fat content by Oil Red O staining

Fat storage was determined in fixed worms using Oil Red O^24^. Oil Red O stain was prepared as a 5 mg/ml stock in isopropanol and equilibrated on a rocker-shaker for 6–7 days. The working solution of Oil Red O was prepared by diluting the equilibrated stock to 60% with water. Stock was mixed thoroughly and filtered using a 0.22 µm filter to remove any particles. Gravid adult worms were treated with sodium hypochlorite to obtain eggs which were grown on respective RNAi plates till L4-YA stage. The worms were washed in 1X M9 to remove any attached bacteria and resuspended in 120 µl 1X PBS. To this an equal volume of 2X MRWB buffer (160 mM KCl, 40 mM NaCl, 14 mM Na_2_EGTA, PIPES pH 7.4, 1 mM spermidine, 0.4 mM spermine, 2% Paraformaldehyde, and 0.2% beta-mercaptoethanol) was added and incubated by shaking for 45 min. The worms were pelleted and washed with 1X PBS. The working solution of Oil Red O was added to the fixed worms and incubated for 1 h on a shaker at room temperature. Following staining, worms were washed thrice with 1X PBS and mounted on 2% agarose slides for visualization using an AxioImager M2 microscope (Carl Zeiss, Germany) fitted with an Axiocam MRc camera. NIH ImageJ software 1.52a was used to quantify fat stores in the worms.

### Oxygen consumption rate (OCR)

Synchronized population of worms was grown at 20 °C till YA stage on RNAi- or OP50-seeded plates from egg onwards. From this population, 400 YA worms were picked into 1X M9 buffer and washed thrice to remove any attached bacteria before placing it in the respiratory chambers of Oxygraph-2k (A&B) (Oroboros Instruments, Innsbruck, Austria). OCR was monitored in real-time as decline in O_2_ saturation in the two chambers. Data, obtained with the inbuilt software Datlab 7, is plotted as the rate of decline in O_2_ consumption (respiration rate) over 10 min of time interval.

### ROS measurement

For measuring intracellular ROS levels, synchronized YA worms were collected and washed thrice in 1X M9 buffer. Worm extracts were prepared by flash freezing worm pellets in liquid nitrogen and freeze-thawing for three cycles, followed by sonication (setting of 30 amplitudes, 12 cycles of 1 s pulse on/off, 5–8 times using Misonix Ultrasonic processor 4000) in 1X PBS. The worm extract was centrifuged at 20,000 × *g* for 15 min at 4 °C, and the protein concentration of the supernatant was determined using Bradford protein estimation kit (Bio-Rad, USA). Supernatant containing 5 μg of protein was pre-incubated with 50 μM of 2,7-dichlorofluorescein diacetate (DCFDA, Molecular Probes, USA) in 100 μL of 1X PBS at 37 °C for 1 h. Fluorescence intensity was measured in FLUOstar Omega (BMG Labtech, USA) every 10 min for 1 h at excitation wavelength 485 nm and emission wavelength 520 nm.

### Pharyngeal pumping

Pharyngeal pumping was counted in L4 worms grown on control RNAi from hatching. A 1-min video of each worm was taken using Axiocam MRm (Carl Zeiss, Germany) camera attached to M205FA microscope (Leica, Germany). The video was slowed down and pharyngeal pumping was counted for this period.

### Autophagy analysis

Transgenic reporter strains expressing GFP-tagged LGG-1 in wild-type, *eat-2(ad465)*, or *sek-1(km4)* mutant background were grown at 20 °C on control, *sek-1*, or *drl-1* RNAi from egg onward. The L3 larval stage worms were picked onto 2% agarose slides and paralyzed using 20 mM of sodium azide. Worms were imaged for LGG-1:GFP puncta in their hypodermal seam cells using AxioImager M2 microscope (Carl Zeiss, Germany) fitted with Axiocam MRm at a magnification of ×630. Scoring of 3–10 seam cells were performed for each of the worms examined. The autophagic events were quantified and plotted as the number of puncta per seam cell by averaging the total number of puncta over all the seam cells counted.

### Total RNA isolation and quantitative real-time PCR

Synchronized L1 worms grown till YA stage on control or *drl-1* RNAi plates were exposed to FUDR to prevent any progeny growth. Post 24 h, worms were collected, washed thrice in 1X M9 buffer and flash-frozen in Trizol (Invitrogen, USA). The worms in Trizol were passed through a freeze–thaw cycle and lysed by vigorous vortexing. RNA was purified by phenol:chloroform:isoamylalcohol extraction followed by ethanol precipitation. The concentration of the RNA was determined using NanoDrop 2000 (Thermo Scientific, USA). The quality of the ribosomal 28 s and 18 s on an agarose gel was used as a measure of integrity. Around 2.5 μg of RNA was used for cDNA synthesis using Superscript III Reverse Transcriptase (Invitrogen, USA). For determining mRNA expression levels, quantitative real-time PCR (qRT-PCR) was performed using the DyNAmo Flash SYBR Green mastermix (Thermo Scientific, USA) and Realplex PCR system (Eppendorf, USA), according to the manufacturer’s specifications. Relative mRNA expression for target genes was determined after normalizing the data to *actin*.

### XDP reporter imaging and analysis

The transgenic *C. elegans* strain carrying a *cyp-35B1* promoter fused to the green fluorescent protein was used to visualize the expression of *cyp-35B1* upon *drl-1* knockdown. CY573 (*bvIs5 [cyp-35B1p::GFP* + *gcy-7p::GFP]*) worms were grown at 20 °C on standard NGM plates seeded with *E. coli* (OP50). Synchronized population of eggs were allowed to hatch and grow till L2 larval stage on plates containing the control or *drl-1* RNAi-seeded bacteria. Post L2, worms were transferred to control, *drl-1* or *sek-1* RNAi plates (Fig. 4b). Worms were imaged using Zeiss Imager M2 at L4-YA stage. Around 50 adult worms were picked onto 2% agarose slides and paralyzed using 50 mM of sodium azide in 1X M9. Worms were imaged using an AxioImager M2 microscope at a magnification of ×100, which was kept similar throughout all conditions. Over 30 transgenic worms per condition were imaged and analyzed. For *fat-2(wa17);Pcyp-35B1::gfp*, the worms were maintained and the experiments performed at 25 °C.

### Fatty acid analysis using GC-MS

To measure fatty acid composition, ~1000 young adult stage worms (containing 0–8 embryos) were washed from feeding plates with water on ice and washed once to remove residual bacteria. After settling on ice again, as much water as possible was removed (~90%). Fatty acids were converted to methyl esters for analysis as previously described^75^. The worm suspensions were incubated for 1 h at 70 °C in 2 ml of 2.5% sulfuric acid in methanol. Following incubation, the reactions were stopped by adding 1 ml of water and then mixed thoroughly with 200 μl of hexane to extract the resulting fatty acid methyl esters. We measured relative amount of fatty acid methyl esters by injecting 2 μl of the hexane layer onto an Agilent 7890 GC/5975C MS in scanning ion mode.

### External supplementation assay

For external supplementation of PUFAs^76^, NGM agar media containing 0.1% Tergitol (NP-40) was autoclaved and allowed to partially cool till the temperature reached 65 °C. To this, 0.3 mM linoleic acid (Catalog no. 90150*)*, 0.5 mM eicasopentaenoic acid (Catalog no. 90110*)* (Cayman Chemical, USA) or ethanol control were added with constant stirring at around 65 °C. After pouring, plates were kept for drying in the dark for 2 days at room temperature. *E. coli* OP50-RNAi strain transformed with control or *sek-1* RNAi plasmid were seeded on these plates. Plates were allowed to dry for 3 days at a temperature of 20 °C. Synchronized population of eggs obtained from sodium hypochlorite treatment of gravid adult CY573 worms (*bvIs5[cyp-35B1p::GFP*+*gcy-7p::GFP]*) were allowed to hatch and grown till YA on different fatty acid supplemented plates at 20 °C. About 36–40 h after YA, worms were imaged using Zeiss Imager M2 as discussed above. At the same time, 50 worms were collected in 1X M9 buffer, washed once with 1X M9 and denatured in 1X Lamelli’s buffer to be used for phospho-western analysis. For oleic acid (OA; catalog no. 01008-1G; Merck, USA), palmitic acid (PA; catalog no. P0500-10G; Merck, USA), stearic acid (SA; catalog no. S4751-10G; Merck, USA), and the fatty acids were supplemented to the bacterial pellet at a concentration of 4.8 mM, respectively. Worms were imaged 24–28 h post YA. For life-span analysis (Fig. 7a, b), supplementation was done from L1 stage.

### Statistical analysis

All survival graphs were plotted using GraphPad Prism 8 (GraphPad Software, Inc., La Jolla, CA). All the statistical analysis to measure *P-*values between survival curves was performed using Log-rank (Mantel–Cox) test through online software OASIS 1.0 [http://sbi.postech.ac.kr/oasis]. Summary of life span containing mean lifespan ± SEM, number of animals (*n*) and *P-*value is provided in Supplementary Table 1. Two-way ANOVA as well as unpaired two-tailed *t* test was used in case of BDR life spans as indicated in the figure legends. Two-way ANOVA was used for ROS (Fig. 3c) and autophagy (Fig. 3e, Supplementary Fig. 3b) measurements. Unpaired two-tailed Student’s *t* test was used for Figs. 3d, 5f, 6a, Supplementary Figs. 1a, b, 2a–c, 3a, 6).

### Western blot analysis

For each sample, 50 worms were picked in 1X M9 buffer, washed twice and collected in 1X SDS lamellae buffer. It was then boiled for 10 min and resolved by 10% SDS-PAGE. After complete run, proteins were transferred onto PVDF membrane (Millipore, Billerica, MA) at 110 V for 1 h at 4 °C in chilled transfer buffer. Following transfer, the membrane was incubated with 5% w/v bovine serum albumin (BSA) prepared in 0.1% TBST for blocking. The membrane was blocked for 1 h at room temperature. Subsequently, primary antibody was added in a 1:5000 dilution, prepared in 0.1% TBST and incubated overnight 4 °C in a rocker-shaker. Next day, after 16 h of incubation, excess primary antibody was washed off with three washes of 0.1% TBST, each wash for 5 min at RT on a rocker-shaker. Secondary antibody (Abcam, Cat no.: ab6721) at a dilution of 1:5000 in 0.1% TBST was added to the blot and incubated for 1 h at RT with rocking. Again, the blot was washed by 0.1% TBST three times, each for 5 min, at RT on a rocker-shaker. Then, the blot was developed using an ECL reagent (Millipore, USA), according to manufacturer’s instructions, to detect antibody binding to specific protein bands on the membrane. Uncropped blots are provided in the Source data file.

Antibody/Supplier name/Catalog no./lot number

1. Phospho-p38-MAPK (Thr180/Tyr182) (3D7) Rabbit mAb/Cell Signaling Technology/9215S/7
2. p38-MAPK Antibody/Cell Signaling Technology/9212S/26
3. β-Actin Antibody/Cell Signaling Technology/4967L/7
4. Goat anti-rabbit IgG H&L (HRP) Secondary antibody/Abcam/ab6721

### Reporting summary

Further information on research design is available in the Nature Research Reporting Summary linked to this article.

## Supplementary information

Supplementary Information

Reporting Summary

## Data Availability

Source data are provided with this paper. All other relevant data are available in the article, supplementary information, or from the corresponding author upon reasonable request. Life-span data used in Figs. 1, 2, 5, 7 and Supplementary Fig. 1 are presented in Supplementary Table 1, along with an independent biological replicate. Source data are provided with this paper.

## Source data

Source Data
