# Supplementary material for: Polyunsaturated fatty acids and p38-MAPK link metabolic reprogramming to cytoprotective gene expression during dietary restriction
Source: Nat Commun. 2020 Sep 25;11:4865. doi: 10.1038/s41467-020-18690-4 (PMC7519657; doi:10.1038/s41467-020-18690-4)
Supplement: Supplementary file 1 — Supplementary Information [file 41467_2020_18690_MOESM1_ESM.pdf]

Supplementary information file

Polyunsaturated fatty acids and p38-MAPK link metabolic reprogramming to cytoprotective gene expression during Dietary Restriction

Chamoli M., Goyala A., Tabrez SS. et al, 2020

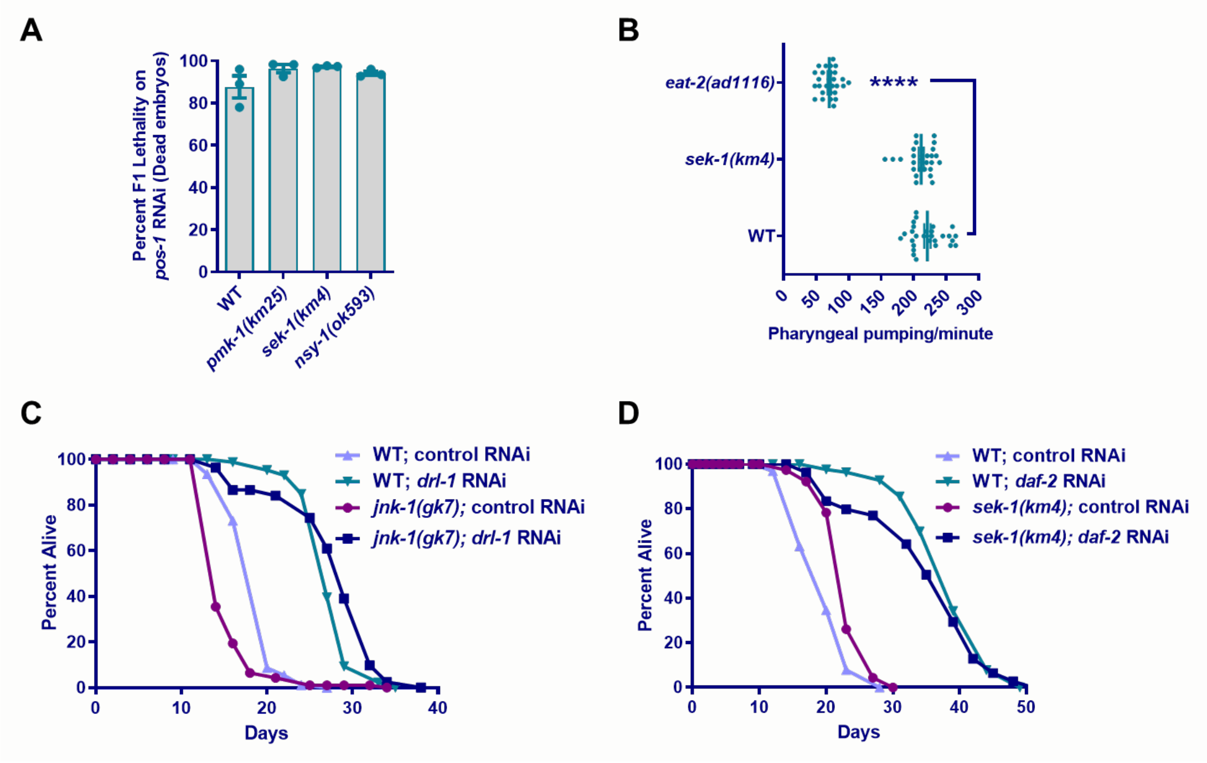

Supplementary Figure 1. (A) The *nsy-1(ok593)*, *sek-1(km4)* and *pmk-1(km25)* mutants are not RNAi defective, similar to wild-type (WT). The worms were grown on *pos-1* RNAi and number of L1 or dead embryos counted. Percent dead embryos plotted on x-axis. n=3 independent experiments. Data presented as mean values ± SEM. **(B)** WT and *sek-1(km4)* have similar pharyngeal pumping rates at L4 stage. The *eat-2(ad1116)* that has slow pumping rate was used as a control. n=28 examined in 2 independent experiments. Unpaired two-tailed *t*-test with Welch's correction, ****P<0.0001. Data represented as mean values ± SEM. **(C)** The knockdown of *drl-1* increases life span in both WT as well as *jnk-1(gk7)*. **(D)** Knocking down *daf-2* using RNAi results in life span extension in both WT as well as in *sek-1(km4)*. Life span and summary data is provided in Supplementary Table 1. Experiments performed at 20 °C. Source data are provided as a Source Data file.

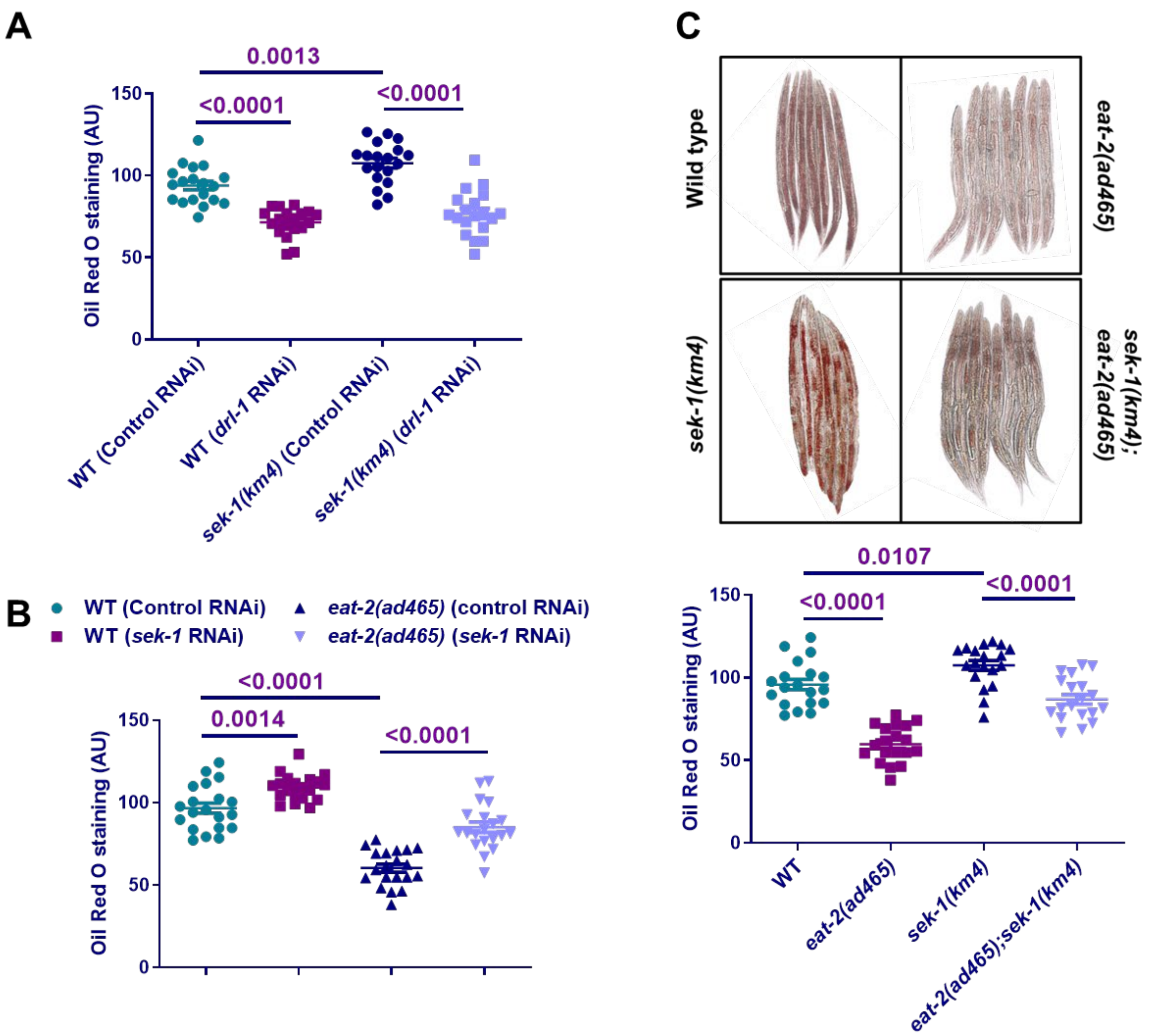

Supplementary Figure 2. (A) Quantification of data shown in Figure 3A. n=19. One of three biologically independent experiments is shown. **(B)** Quantification of data shown in Figure 3B. n=20. One of three biologically independent experiments is shown. **(C)** Lower fat storage was observed in *eat-2(ad465)* and *eat-2(ad465);sek-1(km4)* as compared to WT and *sek-1(km4)*, respectively. Quantification of data is shown below. n=19. One of three biologically independent experiments is shown.

Unpaired two-tailed *t*-test with Welch correction used in all cases. Data are presented as mean values ± SEM. Experiments performed at 20 °C. Source data are provided as a Source Data file.

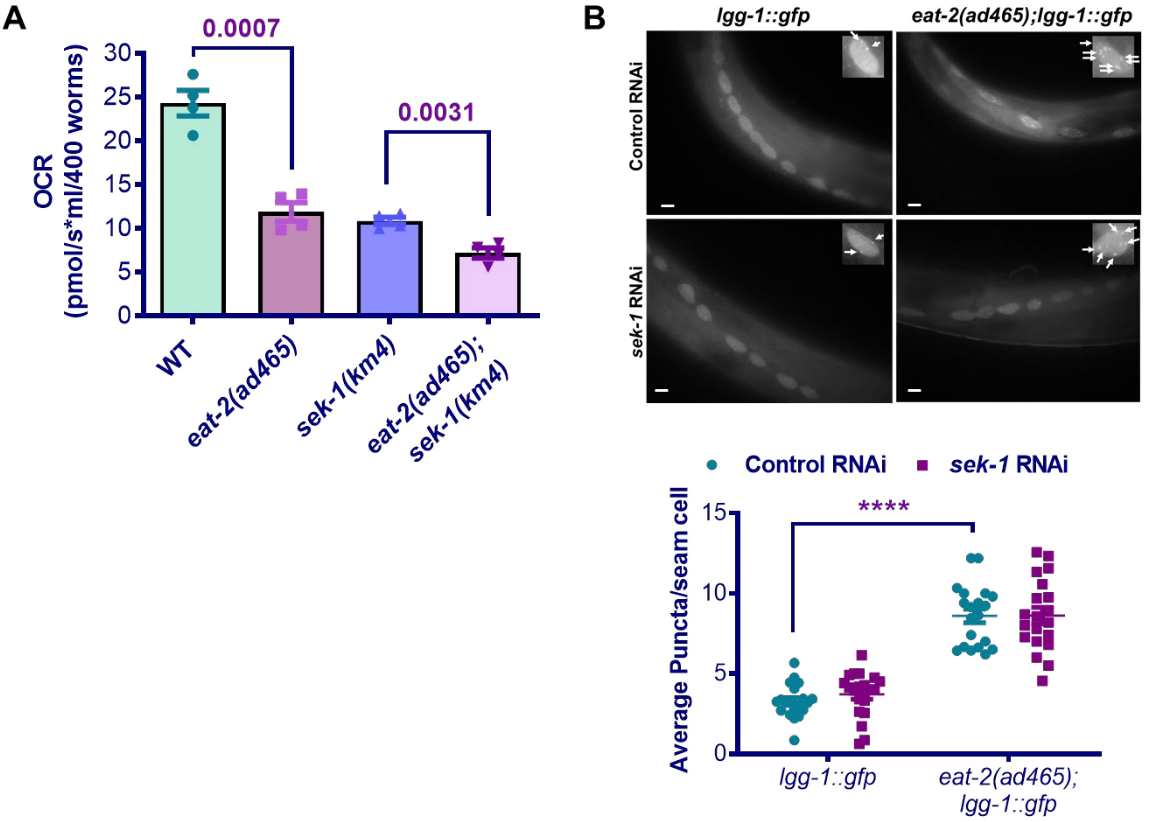

Supplementary Figure 3. (A) The oxygen consumption rate (OCR) is decreased in *eat-2(ad465)* as compared to WT. Similar decrease was observed on comparing *sek-1(km4)* and *eat-2(ad465);sek-1(km4)*. n=4 independent experiments. Data are presented as mean values ± SEM. Unpaired two-tailed *t*-test with Welch's correction. **(B)** Autophagy, as determined by puncta formation in the seam cells of a LGG-1::GFP-expressing strain (upper panel), was increased in *eat-2(ad465)* as compared to WT. Knocking down *sek-1* by RNAi has no effect. Quantification of one of two biologically independent experiments shown (lower panel). n=20. Data are presented as mean values ± SEM. Two-way Annova-Sidak's multiple comparisons test, ****P<0.0001. Scale bar = 10 μm. Experiments performed at 20 °C. Source data are provided as a Source Data file.

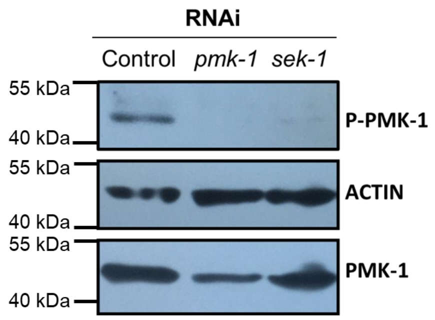

Supplementary Figure 4. Western blot analysis of WT grown on control, *pmk-1* and *sek-1* RNAi. The levels of phospho-PMK-1 is dramatically reduced in all cases. The level of PMK-1 is lower only in case of *pmk-1* RNAi and not in case of *sek-1* RNAi. One of two biologically independent experiments is shown. Experiments were performed at 20 °C. Source data are provided as a Source Data file.

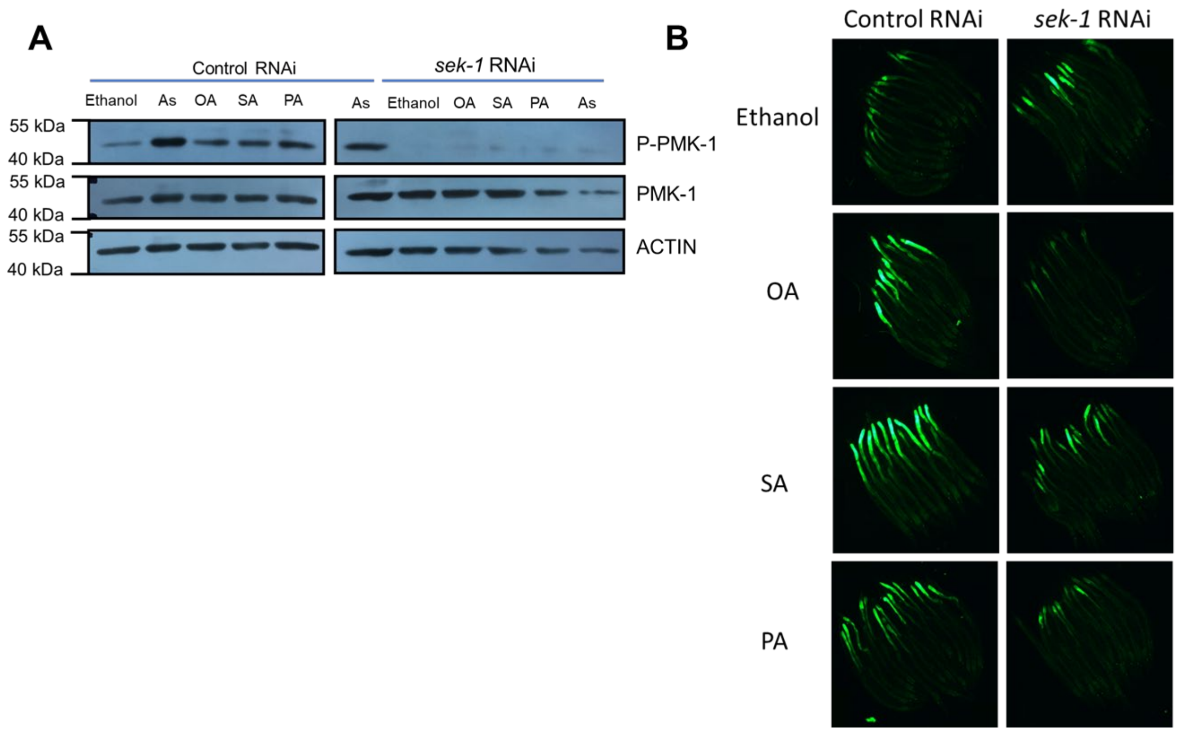

Supplementary Figure 5. (A) Western blot analysis of *Pcyp-35B1*::*gfp* grown on control or *sek-1* RNAi showing that OA, SA and PA supplementation upregulates phospho-PMK-1 levels, in a *sek-1*-dependent manner. Arsenite (As) treatment of worms was taken as a positive control. One of two biologically independent experiments is shown. **(B)** External supplementation of oleic acid (OA), stearic acid (SA) and palmitic acid (PA) induces expression of GFP (at 30-36 hours post YA) in the *Pcyp-35B1*::*gfp* worms that was suppressed when *sek-1* is knocked down using RNAi. Data from one of two biologically independent experiments shown. Multiple overlapping images were acquired at 100X magnification to cover the entire worm body and stitched together to generate a contiguous image. Experiments were performed at 20 °C. Source data are provided as a Source Data file.

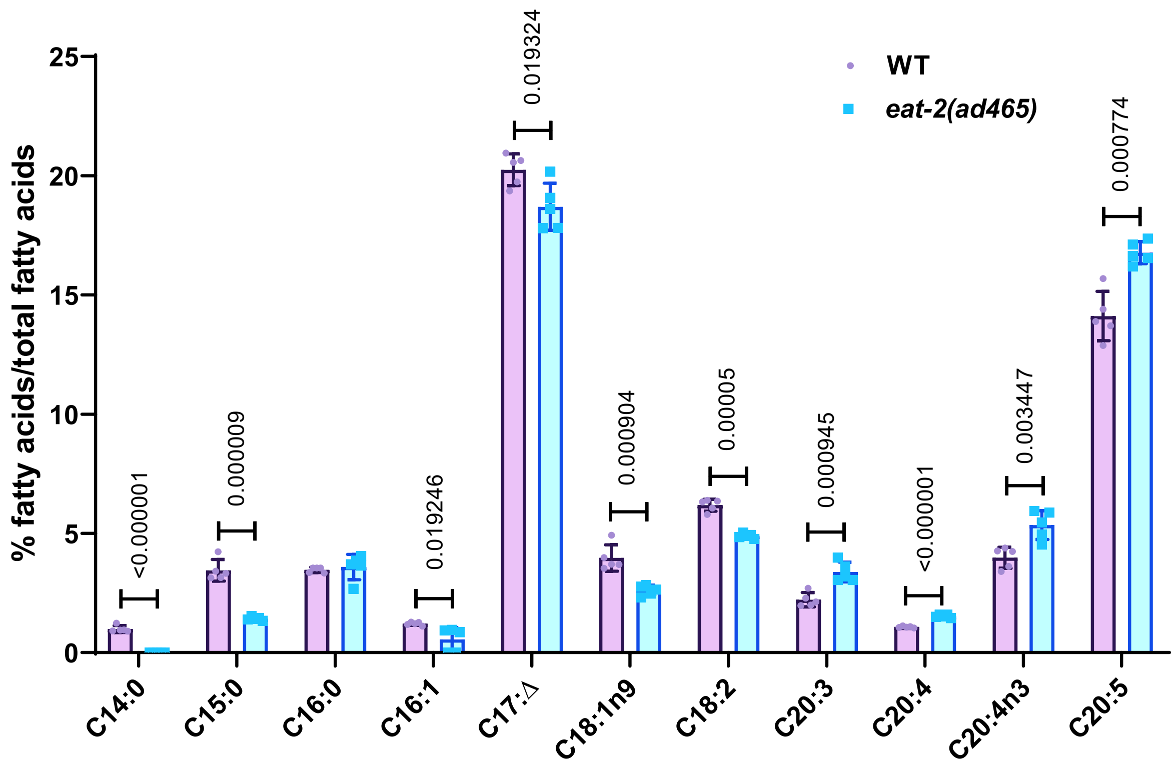

Supplementary Figure 6. GC-MS analysis revealed that PUFAs are differentially regulated in *eat-2(ad465)* as compared to WT. n=5 biologically independent samples. Data are presented as mean values ± Std. Dev. Unpaired two-tailed *t*-test. Experiments were performed at 20 °C. Source data are provided as a Source Data file.

Supplementary Table 1: Summary of life span analysis, related to Figures 1, 2, 5 and 7, Supplementary Figure 1.

Genetic Background	RNAi used[a]	Mean ± SEM (Days)	n	% change with respect to Control	*P*-value		Genetic Background	RNAi used[a]	Mean ± SEM (Days)	n	% change with respect to Control	*P*-value
Set1 (Figure 1A, B)							Set2					
**WT**	**Control**	19.6 ± 0.42	68				**WT**	**Control**	18.21 ± 0.33	85		
**WT**	***drl-1***	30.44 ± 0.33	104	55.31	<0.0001		**WT**	***drl-1***	27.9 ± 0.64	50	53.21	<0.0001
pmk-1(km25)	**Control**	18.93 ± 0.63	42				***pmk-1(km25)***	**Control**	15.61 ± 0.26	99		
pmk-1(km25)	***drl-1***	17.76 ± 0.53	86	-6.18	0.1793		***pmk-1(km25)***	***drl-1***	18.65 ± 0.53	79	19.47	<0.0001
Set1 (Figure 1A, C)							Set2					
**WT**	**Control**	19.6 ± 0.42	68				**WT**	**Control**	16.56 ± 0.29	97		
**WT**	***drl-1***	30.44 ± 0.33	104	55.31	<0.0001		**WT**	***drl-1***	30.38 ± 0.29	97	83.45	<0.0001
nsy-1(ok593)	**Control**	24.41 ± 0.36	128				***nsy-1(ok593)***	**Control**	24.52 ± 0.57	86		
nsy-1(ok593)	***drl-1***	26.27 ± 0.48	102	7.62	0.0003		***nsy-1(ok593)***	***drl-1***	26.4 ± 0.81	75	7.67	0.0071
Set1 (Figure 1D)							Set2					
**WT**	**Control**	17.49 ± 0.3	88				**WT**	**Control**	19.6 ± 0.42	68		
**WT**	***drl-1***	30.29 ± 0.5	73	73.18	<0.0001		**WT**	***drl-1***	30.44 ± 0.33	104	55.31	<0.0001
sek-1(km4)	**Control**	18.25 ± 0.35	81				***sek-1(km4)***	**Control**	21.71 ± 0.47	63		
sek-1(km4)	***drl-1***	18.81 ± 0.49	78	3.07	0.2051		***sek-1(km4)***	***drl-1***	16.63 ± 0.51	81	-23.40	<0.0001
Set1 (Supplementary Figure 1C)												
**WT**	**Control**	19.02± 0.27	93									
**WT**	***drl-1***	27.48 ± 0.33	86	44.47	<0.0001							
jnk-1(gk7)	**Control**	15.56 ± 0.32	93									
jnk-1(gk7)	***drl-1***	27.79 ± 0.64	82	78.59	<0.0001							

Set1 (Supplementary Figure 1D)							Set2					
**WT**	**Control**	19.84 ± 0.25	228				**WT**	**Control**	18.88 ± 0.34	105		
**WT**	***daf-2***	38.7 ± 0.49	166	95.06	<0.0001		**WT**	***daf-2***	36.71 ± 0.41	156	94.44	<0.0001
sek-1(km4)	**Control**	23.21 ± 0.31	115				***sek-1(km4)***	**Control**	25.25 ± 0.41	122		
sek-1(km4)	***daf-2***	34.93 ± 0.86	109	50.50	<0.0001		***sek-1(km4)***	***daf-2***	34.02 ± 0.68	154	34.73	<0.0001
Set1 (Figure 2A)**							Set2**					
**WT**	**Control**	19.84 ± 0.25	228				**WT**	**Control**	18.88 ± 0.34	105		
**WT**	***sek-1***	22.13 ± 0.41	132	11.54	<0.0001		**WT**	***sek-1***	23.31 ± 0.33	153	23.46	<0.0001
eat-2(ad465)	**Control**	35.77 ± 0.39	181	80.29	<0.0001		***eat-2(ad465)***	**Control**	32.28 ± 0.43	160	70.97	<0.0001
eat-2(ad465)	***sek-1***	23.16 ± 0.36	204	-35.25	<0.0001		***eat-2(ad465)***	***sek-1***	27.19 ± 0.48	154	-15.77	<0.0001
Set1 (Figure 2B)**							Set2**					
**WT**	**Control**	19.84 ± 0.25	228				**WT**	**Control**	18.88 ± 0.34	105		
sek-1(km4)	**Control**	22.44 ± 0.3	130	13.10	<0.0001		***sek-1(km4)***	**Control**	24.61 ± 0.37	122	30.35	<0.0001
eat-2(ad465)	**Control**	35.77 ± 0.39	181	80.29	<0.0001		***eat-2(ad465)***	**Control**	32.28 ± 0.43	160	70.97	<0.0001
eat-2(ad465); sek-1(km4)	**Control**	26.17 ± 0.52	115	-26.84	<0.0001		***eat-2(ad465); sek-1(km4)***	**Control**	22.18 ± 0.52	121	-31.29	<0.0001
**These experiments were set up together and have same controls							**These experiments were set up together and have same controls					
Set1 (Figure 2C - Mean of four sets)	**OP50-L44440**						Set2	**OP50-L44440**				
**WT**	**OD 3.0**	31 ± 0.67	43				**WT**	**OD 3.0**	30.11 ± 0.78	45		
**WT**	**OD 1.0**	36.04 ± 0.9	46	16.26	<0.0001		**WT**	**OD 1.0**	34.27 ± 1.14	41	13.82	0.0003
**WT**	**OD 0.5**	40.95 ± 1.15	44	32.10	<0.0001		**WT**	**OD 0.5**	35.05 ± 0.93	43	16.41	<0.0001
**WT**	**OD 0.25**	42.07 ± 1.35	44	35.71	<0.0001		**WT**	**OD 0.25**	36 ± 1.32	43	19.56	<0.0001
**WT**	**OD 0.125**	40.36 ± 1.6	44	30.19	<0.0001		**WT**	**OD 0.125**	37.28 ± 1.2	46	23.81	<0.0001
**WT**	**OD 0.0156**	35.43 ± 1.37	46	14.29	0.0001		**WT**	**OD 0.0156**	31.55 ± 0.96	47	4.78	0.1039

sek-1(km4)	OD 3.0	27.75 ± 1	48				*sek-1(km4)*	OD 3.0	25.65 ± 0.64	40		
sek-1(km4)	OD 1.0	27.02 ± 1.03	45	-2.63	0.6280		*sek-1(km4)*	OD 1.0	25.67 ± 0.83	48	0.08	0.4368
sek-1(km4)	OD 0.5	25.19 ± 1.05	47	-9.23	0.1053		*sek-1(km4)*	OD 0.5	25.25 ± 1.01	44	-1.56	0.3645
sek-1(km4)	OD 0.25	24.72 ± 1.06	43	-10.92	0.0463		*sek-1(km4)*	OD 0.25	25.41 ± 1.06	46	-0.94	0.2710
sek-1(km4)	OD 0.125	23.07 ± 0.95	41	-16.86	0.0012		*sek-1(km4)*	OD 0.125	24.09 ± 0.93	47	-6.08	0.6502
sek-1(km4)	OD 0.0156	24.13 ± 0.98	45	-13.05	0.0121		*sek-1(km4)*	OD 0.0156	25.84 ± 0.76	49	0.74	0.4126
Set3	OP50-L44440						Set4	OP50-L44440				
WT	OD 3.0	21.69 ± 0.54	42				WT	OD 3.0	23.06 ± 0.72	47		
WT	OD 1.0	28.79 ± 1.1	47	32.73	<0.0001		WT	OD 1.0	30.64 ± 0.73	44	32.87	<0.0001
WT	OD 0.5	34.77 ± 1.47	47	60.30	<0.0001		WT	OD 0.5	34.13 ± 1.41	45	48.01	<0.0001
WT	OD 0.25	33.89 ± 1.51	44	56.25	<0.0001		WT	OD 0.25	27.36 ± 1.15	45	18.65	0.0002
WT	OD 0.125	30.25 ± 1.84	32	39.47	<0.0001		WT	OD 0.125	27.17 ± 1.02	41	17.82	0.0007
WT	OD 0.0156	24.8 ± 1.18	45	14.34	0.0192		WT	OD 0.0156	22.19 ± 0.91	43	-3.77	0.7390
sek-1(km4)	OD 3.0	22.34 ± 0.85	35				*sek-1(km4)*	OD 3.0	23.14 ± 1.22	37		
sek-1(km4)	OD 1.0	24.95 ± 0.9	37	11.68	0.0170		*sek-1(km4)*	OD 1.0	20.23 ± 1.11	39	-12.58	0.0752
sek-1(km4)	OD 0.5	23.83 ± 1.09	47	6.67	0.0767		*sek-1(km4)*	OD 0.5	20.1 ± 0.81	41	-13.14	0.0098
sek-1(km4)	OD 0.25	22.61 ± 0.99	44	1.21	0.5775		*sek-1(km4)*	OD 0.25	20.4 ± 1.13	30	-11.84	0.0424
sek-1(km4)	OD 0.125	24.2 ± 0.9	46	8.33	0.0807		*sek-1(km4)*	OD 0.125	18.78 ± 0.95	45	-18.84	0.0046
sek-1(km4)	OD 0.0156	24.72 ± 1.03	43	10.65	0.0365		*sek-1(km4)*	OD 0.0156	20.05 ± 1.19	38	-13.35	0.0704
Set1 (Figure 2D)	HT115-L44440						Set2	HT115-L44440				
WT	control	18.99 ± 0.25	202				WT	control	18.01 ± 0.19	175		
WT	2-DOG	23.37 ± 0.44	79	23.06	<0.0001		WT	2-DOG	22.4 ± 0.38	151	24.37534703	<0.0001
sek-1(km4)	control	22.36 ± 0.51	113				*sek-1(km4)*	control	22.22 ± 0.5	113		

sek-1(km4)	2-DOG	26.97 ± 0.43	116	20.62	<0.0001		sek-1(km4)	2-DOG	25.9 ± 0.49	124	16.56165617	<0.0001
Set1 (Figure 5B)	RNAi used[a]						Set2	RNAi used[a]				
WT	Control	12.92 ± 0.16	102				WT	Control	15.65 ± 0.13	231		
	drl-1	15.07 ± 0.17	213	16.64	<0.0001			drl-1	18.11 ± 0.1	417	15.72	<0.0001
fat-6(tm331); fat-7(wa36)	Control	11.82 ± 0.15	130				fat-6(tm331); fat-7(wa36)	Control	14.35 ± 0.09	244		
	drl-1	11.67 ± 0.16	196	-1.27	0.7346			drl-1	15.42 ± 0.14	237	7.46	<0.0001
Set1 (Figure 5C)							Set2					
WT	Control	12.89 ± 0.07	428				WT	Control	14.54 ± 0.15	242		
	drl-1	14.99 ± 0.17	193	16.29	<0.0001			drl-1	17.11 ± 0.18	292	17.67537827	<0.0001
fat-2(wa17)	Control	13.39 ± 0.27	51				fat-2(wa17)	Control	15.49 ± 0.41	59		
	drl-1	12.38 ± 0.24	63	-7.54	0.0066			drl-1	15.5 ± 0.29	92	0.064557779	0.9096
Set1 (Figure 5D)							Set2					
WT	Control	13.70 ± 0.21	80				WT	Control	14.40 ± 0.22	65		
fat-2(tm789)	Control	14.79 ± 0.14	190	7.96	<0.0001		fat-2(tm789)	Control	14.36 ± 0.21	125	-0.28	0.6358
eat-2(ad465)	Control	17.33 ± 0.12	201	26.50	<0.0001		eat-2(ad465)	Control	19.11 ± 0.16	74	32.71	<0.0001
eat-2(ad465); fat-2(tm789)	Control	15.48 ± 0.17	120	-10.68	<0.0001		eat-2(ad465); fat-2(tm789)	Control	15.34 ± 0.28	56	-19.73	<0.0001
Set1 (Figure 5E -Mean of two sets)	HT115-L44440						Set2	HT115-L44440				
WT	OD 3.0	14.14 ± 0.22	42				WT	OD 3.0	14.11 ± 0.32	38		
WT	OD 1.0	18.27 ± 0.29	41	29.21	<0.0001		WT	OD 1.0	16.21 ± 0.52	43	14.88	0.0005
WT	OD 0.5	18.56 ± 0.38	41	31.26	<0.0001		WT	OD 0.5	18.67 ± 0.48	45	32.32	<0.0001
WT	OD 0.25	19.86 ± 0.37	42	40.45	<0.0001		WT	OD 0.25	18.80 ± 0.41	46	33.24	<0.0001
WT	OD 0.125	18.45 ± 0.42	38	30.48	<0.0001		WT	OD 0.125	17.17 ± 0.47	42	21.69	<0.0001

**WT**	**OD 0.0156**	17.20 ± 0.42	40	21.64	<0.0001		**WT**	**OD 0.0156**	13.57 ± 0.49	42	-3.83	0.3848
fat-2(wa17)	**OD 3.0**	14.67 ± 0.36	45				***fat-2(wa17)***	**OD 3.0**	15.37 ± 0.44	46		
fat-2(wa17)	**OD 1.0**	16.87 ± 0.41	46	15.00	0.0001		***fat-2(wa17)***	**OD 1.0**	15.98 ± 0.47	42	3.97	0.3056
fat-2(wa17)	**OD 0.5**	15.86 ± 0.43	42	8.11	0.0335		***fat-2(wa17)***	**OD 0.5**	16.23 ± 0.55	48	5.60	0.0731
fat-2(wa17)	**OD 0.25**	17.21 ± 0.32	42	17.31	<0.0001		***fat-2(wa17)***	**OD 0.25**	15.17 ± 0.57	47	-1.30	0.6709
fat-2(wa17)	**OD 0.125**	16.15 ± 0.45	41	10.09	0.0061		***fat-2(wa17)***	**OD 0.125**	15.71 ± 0.56	42	2.21	0.2922
fat-2(wa17)	**OD 0.0156**	15.32 ± 0.60	38	4.43	0.117		***fat-2(wa17)***	**OD 0.0156**	14.98 ± 0.44	47	-2.54	0.4834
Set1 (Figure 7A, B)	**RNAi used[a]**						Set2	**RNAi used[a]**				
**WT + Ethanol**	**Control**	11.30 ± 0.07	270				**WT + Ethanol**	**Control**	12.27 ± 0.09	212		
	drl-1	15.14 ± 0.10	401	33.98	<0.0001			***drl-1***	13.81 ± 0.12	138	12.55	<0.0001
***fat-2(wa17)* + Ethanol**	**Control**	07.62 ± 0.17	126				***fat-2(wa17)* + Ethanol**	**Control**	08.75 ± 0.25	59		
	drl-1	08.01 ± 0.13	201	5.12	0.1184			***drl-1***	07.29 ± 0.16	92	-16.69	<0.0001
**WT + EPA**	**Control**	08.92 ± 0.13	128				**WT + EPA**	**Control**	11.30 ± 0.09	257		
	drl-1	12.74 ± 0.08	313	42.83	<0.0001			***drl-1***	14.14 ± 0.10	228	25.13	<0.0001
***fat-2(wa17)* + EPA**	**Control**	09.22 ± 0.26	114				***fat-2(wa17)* + EPA**	**Control**	09.70 ± 0.27	46		
	drl-1	12.81 ± 0.23	113	38.94	<0.0001			***drl-1***	13.36 ± 0.26	74	37.73	<0.0001
**WT + LA**	**Control**	11.93 ± 0.09	161				**WT + LA**	**Control**	12.19 ± 0.11	178		
	drl-1	13.61 ± 0.10	310	14.08	<0.0001			***drl-1***	14.16 ± 0.11	192	16.16	<0.0001
***fat-2(wa17)* + LA**	**Control**	10.81 ± 0.19	145				***fat-2(wa17)* + LA**	**Control**	11.27 ± 0.20	71		
	drl-1	14.92 ± 0.25	114	38.02	<0.0001			***drl-1***	14.33 ± 0.39	49	27.15	<0.0001

[a] All RNAi were taken from the Ahringer RNAi library, unless otherwise mentioned

Survival graphs were plotted using GraphPad Prism 8 (GraphPad Software, Inc., La Jolla, CA). All the statistical analysis to measure *P*-values between survival curves was performed using Log-rank (Mantel-Cox) test through online software OASIS 1.0 (http://sbi.postech.ac.kr/oasis). Data is represented as mean lifespan ± SEM. number of animals = n. Conditions for all the lifespans experiments are provided in Figure Legends.

Supplementary Table 2, related to Figure 4, 5: List of primers used in the study.

Gene name (Target)	Primer Name	Sequence
**qRT-Primers**		
**Cytoprotective (CyTP) xenobiotic detoxification genes**		
cyp-33C8	Forward Primer	CGCTGGATGATGTGCTCAACTACTGG
	Reverse Primer	GCTTCTTCTGCTCTTTCAGGTAGG
cyp-34A4	Forward Primer	GATTTGAACAGGGTGACCCAGAAT
	Reverse Primer	TCGATGACATGCTCACCACT
cyp-32B1	Forward Primer	GGTGTGTTGAAGTTATGGTTGGGACC
	Reverse Primer	TGTCGCCGGTGCTGATTAAAAGAC
ugt-16	Forward Primer	CTTGCTGACGATCGACTAACC
	Reverse Primer	CGGTCTGTATGGCTTCTCTAAG
nhr-31	Forward Primer	GAGTTGTGAAAGTTGAAAGAGTTCC
	Reverse Primer	CTCCATTCTGTGATCCACCACT
nhr-57	Forward Primer	CCGGAAGTTGTTCAAGCAATCC
	Reverse Primer	GTCATAGTCACCGAGTTCCAGA
nhr-206	Forward Primer	ATCCAGCTGTCTCCGATTTTCC
	Reverse Primer	GATCAGCACCGTGAATCTGT
ftn-1	Forward Primer	GAGTGGGGAACTGTCCTTGA
	Reverse Primer	GATCGAATGTACCTGCTCTTCC
pgp-9	Forward Primer	TACAGGCTTCATGCTTCATGG
	Reverse Primer	ACTGAGCCATCATCTGG
cyp-35B1	Forward Primer	CTTCATGTCAGTAATAATCTTGG
	Reverse Primer	CAATTTCGGCACATCTCGTG
ugt-50	Forward Primer	GATATGTGTGCAGATCTACTTGG
	Reverse Primer	GTTGAACAACCTCACTATAG
gst-6	Forward Primer	CAAAAATAACACTCCATTC
	Reverse Primer	GCCGCCTCGGTGTCATTTTGTC
gst-19	Forward Primer	GAAGTCAAAGTCCCCAATG
	Reverse Primer	CAGCAAATCCGAATTTCAGAG
act-1	Forward Primer	CTCTTGCCCCATCAACCATG
	Reverse Primer	CTTGCTTGGAGATCCACATC
**Primers used to confirm the p38-MAPK deletion strains used in the study**		
sek-1(km4)	WT Forward Primer	GGATTTCAAACGCAGGTCACTCGT
	WT Reverse Primer	CCGCGTCACAGACTGTTCT
	Mutant Reverse Primer	CGGTTGACTCGGAAAGAAAC
pmk-1(km25)	WT Forward Primer	CCATGACCTCAGAGCCTCTTT
	WT Reverse Primer	CTCGTGGAGTCGGATGAAGT
	Mutant Reverse Primer	TCAACAGTCTGCGTGTAATGC
nsy-1(ok593)	WT Forward Primer	TCTGGAAAACAGCCAACA
	WT Reverse Primer	CTCGTGCAGCGTACACAGTT
	Mutant Reverse Primer	CAATCCACGTAGCCAACTGA
