# Supplementary material for: Polyunsaturated fatty acids and p38-MAPK link metabolic reprogramming to cytoprotective gene expression during dietary restriction
Source: Nat Commun. 2020 Sep 25;11:4865. doi: 10.1038/s41467-020-18690-4 (PMC7519657; doi:10.1038/s41467-020-18690-4)
Supplement: Supplementary file 2 — Reporting Summary [file 41467_2020_18690_MOESM2_ESM.pdf]

nature research

Corresponding author(s): Arnab Mukhopadhyay

Last updated by author(s): 2020/22/08

Reporting Summary

Nature Research wishes to improve the reproducibility of the work that we publish. This form provides structure for consistency and transparency in reporting. For further information on Nature Research policies, see Authors & Referees and the Editorial Policy Checklist .

Statistics

For all statistical analyses, confirm that the following items are present in the figure legend, table legend, main text, or Methods section.

n/a	Confirmed	
☐	☒	The exact sample size (n) for each experimental group/condition, given as a discrete number and unit of measurement
☐	☒	A statement on whether measurements were taken from distinct samples or whether the same sample was measured repeatedly
☐	☒	The statistical test(s) used AND whether they are one- or two-sided<br>*Only common tests should be described solely by name; describe more complex techniques in the Methods section.*
☒	☐	A description of all covariates tested
☒	☐	A description of any assumptions or corrections, such as tests of normality and adjustment for multiple comparisons
☐	☒	A full description of the statistical parameters including central tendency (e.g. means) or other basic estimates (e.g. regression coefficient) AND variation (e.g. standard deviation) or associated estimates of uncertainty (e.g. confidence intervals)
☐	☒	For null hypothesis testing, the test statistic (e.g. F, t, r) with confidence intervals, effect sizes, degrees of freedom and P value noted<br>*Give P values as exact values whenever suitable.*
☒	☐	For Bayesian analysis, information on the choice of priors and Markov chain Monte Carlo settings
☒	☐	For hierarchical and complex designs, identification of the appropriate level for tests and full reporting of outcomes
☒	☐	Estimates of effect sizes (e.g. Cohen's d, Pearson's r), indicating how they were calculated

Our web collection on statistics for biologists contains articles on many of the points above.

Software and code

Policy information about availability of computer code

Data collection	AxioVision Rel. 4.8, Mastercycler ep RealPlex, Oroboros DatLab 7, Quantity One 4.6.9
Data analysis	GraphPad Prism 8 (GraphPad Software, Inc.,La Jolla, CA). All the statistical analysis to measure P-values between survival curves was performed using Log-rank (Mantel-Cox) test through online software OASIS 1.0 (http://sbi.postech.ac.kr/oasis). ImageJ 1.52a (NIH) Software.

For manuscripts utilizing custom algorithms or software that are central to the research but not yet described in published literature, software must be made available to editors/reviewers. We strongly encourage code deposition in a community repository (e.g. GitHub). See the Nature Research guidelines for submitting code & software for further information.

Data

Policy information about availability of data

All manuscripts must include a data availability statement. This statement should provide the following information, where applicable:

- Accession codes, unique identifiers, or web links for publicly available datasets
- A list of figures that have associated raw data
- A description of any restrictions on data availability

There are no accession codes, unique identifiers or weblinks in our study and no restrictions on data availability. All relevant data are available from the authors on reasonable request. Life span data used in Figures 1, 2, 5, 7 and Supplementary Figure 1 is presented in Supplementary Table 1, along with an independent biological replicate. The following figures/panels have data associated with them that is provided as an excel 'Source' file: Figures 3, 4, 5, 6, Supplementary Figures 1, 2, 3 and 6.

Field-specific reporting

Please select the one below that is the best fit for your research. If you are not sure, read the appropriate sections before making your selection.

- [x] Life sciences
- [ ] Behavioural & social sciences
- [ ] Ecological, evolutionary & environmental sciences

For a reference copy of the document with all sections, see nature.com/documents/nr-reporting-summary-flat.pdf

Life sciences study design

All studies must disclose on these points even when the disclosure is negative.

Sample size

No statistical methods were applied to predetermine sample size. However, sample sizes reported here are consistent to data presented in previous publications (doi:10.1016/j.cub.2011.07.042; doi:10.1371/journal.pone.0012810; doi:10.1371/journal.pone.0028417.g006; doi:10.1038/nature05837; doi.org/10.1371/journal.pgen.1007608.; 10.1038/s41467-017-00370-5; 10.1111/acel.12477; 10.1111/acel.12218)

Data exclusions

None

Replication

To ensure reproducibility multiple biological replicates of each experiments were conducted. Number of repeats performed for each experiments are included in the method and figures legend. Experiments were replicated by multiple individuals in the lab who are authors in the manuscript.

Randomization

Worms were randomly allocated into different experimental groups by transferring from a single population and into different plates.

Blinding

Investigators were not blinded. Most experiments, including life spans, fat storage and fluorescence imaging were conducted by multiple investigators to confirm results. Fatty acid analysis is based on the quantitative GCMS where sample preparation, data collection and analysis were performed by different persons. For imaging experiments, the images were acquired, processed and analyzed by commercial software. Data collection and analysis for QRT-PCR analysis was performed using commercial software. Experiments were conducted based on random sampling and reasonably large sample sizes.

Reporting for specific materials, systems and methods

We require information from authors about some types of materials, experimental systems and methods used in many studies. Here, indicate whether each material, system or method listed is relevant to your study. If you are not sure if a list item applies to your research, read the appropriate section before selecting a response.

Materials & experimental systems

n/a	Involved in the study	
☐	☒	Antibodies
☒	☐	Eukaryotic cell lines
☒	☐	Palaeontology
☐	☒	Animals and other organisms
☒	☐	Human research participants
☒	☐	Clinical data

Methods

n/a	Involved in the study	
☒	☐	ChIP-seq
☒	☐	Flow cytometry
☒	☐	MRI-based neuroimaging

Antibodies

Antibodies used

Antibody/Supplier name/ Catalog no./lot number
1. Phospho-p38 MAPK (Thr180/Tyr182) (3D7) Rabbit mAb/Cell Signalling Technology/9215S/7
2. p38 MAPK Antibody/Cell Signalling Technology/9212S/26
3. β-Actin Antibody/Cell Signalling Technology/4967L/7
4. Goat Anti-Rabbit IgG H&L secondary antibody (HRP)/Abcam/ab6721

Validation

All antibodies used in the study are commercially validated in multiple model systems. These antibodies have been used in C. elegans in the studies listed below:
1. Phospho-p38 MAPK/Cell Signalling Technology/9215S- (https://doi: 10.1111/j.1474-9726.2012.00829.x; https://doi.org/10.1371/journal.pgen.1007608)
2. p38 MAPK Antibody/Cell Signalling Technology/9212S- (https://doi.org/10.1038/ncomms4563; https://doi.org/10.1371/journal.pgen.1007608)
3. β-Actin Antibody/Cell Signalling Technology/4967L- (https://doi.org/10.1016/j.celrep.2019.06.078; https://doi.org/10.1371/journal.pgen.1007608)

Animals and other organisms

Policy information about studies involving animals; ARRIVE guidelines recommended for reporting animal research

Laboratory animals	Caenorhabditis elegans. The strains used in the study are: N2 Bristol as wild-type, VC390 nsy-1(ok593) II, KU4 sek-1(km4) X, KU25 pmk-1(km25) IV, VC8 jnk-1(gk7) IV, DA465 eat-2(ad465) II, DA1116 eat-2(ad1116) II, CY573 bvIs5[cyp-35B1p::GFP + gcy-7p::GFP], BX26 fat-2(wa17) IV, BX156 fat-6(tm331) IV;fat-7(wa36) V, DA2123 adIs2122 [lgg-1p::GFP::lgg-1 + rol-6(su1006)], fat-2(tm789) IV. The strains eat-2(ad465);fat-2(tm789), eat-2(ad465) II;sek-1(km4) X and sek-1(km4);adIs2122 were generated in the lab. Stages used are L1, L3, L4, young adults and gravid adults for the studies. Individual stages are mentioned when referring to the experiments in the manuscript.
Wild animals	No wild animals were used in this study.
Field-collected samples	No field-collected samples were used in this study.
Ethics oversight	No ethical oversight or guidance was required. There were no vertebrates or humans included in the study.

Note that full information on the approval of the study protocol must also be provided in the manuscript.
